# Evaluation of the polyphenolic profile of native Ecuadorian stingless bee honeys (*Tribe: Meliponini*) and their antibiofilm activity on susceptible and multidrug-resistant pathogens: An exploratory analysis

**DOI:** 10.1016/j.crfs.2023.100543

**Published:** 2023-06-29

**Authors:** Fausto Sebastián Cabezas-Mera, María Belén Atiencia-Carrera, Irina Villacrés-Granda, Adrian Alexander Proaño, Alexis Debut, Karla Vizuete, Lorena Herrero-Bayo, Ana M. Gonzalez-Paramás, Francesca Giampieri, Reinier Abreu-Naranjo, Eduardo Tejera, José M. Álvarez-Suarez, António Machado

**Affiliations:** a Universidad San Francisco de Quito USFQ, Colegio de Ciencias Biológicas y Ambientales COCIBA, Instituto de Microbiología, Laboratorio de Bacteriología, Calle Diego de Robles y Pampite, Quito, 170901, Ecuador; b Programa de Doctorado Interuniversitario en Ciencias de la Salud, Universidad de Sevilla, Sevilla, Spain; c Facultad de Ingeniería y Ciencias Agropecuarias Aplicadas, Grupo de Bioquimioinformática, Universidad de Las Américas (UDLA), De Los Colimes esq, Quito, 170513, Quito, Ecuador; d Laboratorios de Investigación, Universidad de Las Américas (UDLA), Vía a Nayón, Quito, 170124, Ecuador; e Departamento de Ciencias de la Vida y la Agricultura, Universidad de las Fuerzas Armadas ESPE, Sangolquí, 171103, Ecuador; f Centro de Nanociencia y Nanotecnología, Universidad de Las Fuerzas Armadas ESPE, Sangolquí, 171103, Ecuador; g Grupo de Investigación en Polifenoles (GIP-USAL), Universidad de Salamanca, Campus Miguel de Unamuno, 37008, Salamanca, Spain; h Research Group on Food, Nutritional Biochemistry and Health, Universidad Europea del Atlántico, C. Isabel Torres, 21, 39011, Santander, Cantabria, Spain; i Departamento de Ciencias de La Vida, Universidad Estatal Amazónica, Puyo, 160150, Ecuador; j Universidad San Francisco de Quito USFQ, Colegio de Ciencias e Ingenierías, Departamento de Ingeniería en Alimentos, Calle Diego de Robles y Pampite, Quito, 170901, Ecuador

**Keywords:** Antibiofilm agents, Pot-honey, Meliponini, Stingless bees, Antimicrobial activity

## Abstract

Biofilms are associated with infections that are resistant to conventional therapies, contributing to the antimicrobial resistance crisis. The need for alternative approaches against biofilms is well-known. Although natural products like stingless bee honeys (tribe: *Meliponini*) constitute an alternative treatment, much is still unknown. Our main goal was to evaluate the antibiofilm activity of stingless bee honey samples against multidrug-resistant (MDR) pathogens through biomass assays, fluorescence (cell count and viability), and scanning electron (structural composition) microscopy. We analyzed thirty-five honey samples at 15% (v/v) produced by ten different stingless bee species (*Cephalotrigona* sp., *Melipona* sp., *M. cramptoni*, *M. fuscopilosa*, *M. grandis*, *M. indecisa*, *M. mimetica*, *M. nigrifacies*, *Scaptotrigona problanca*, and *Tetragonisca angustula*) from five provinces of Ecuador (Tungurahua, Pastaza, El Oro, Los Ríos, and Loja) against 24-h biofilms of *Staphylococcus aureus*, *Klebsiella pneumoniae*, *Candida albicans*, and *Candida tropicalis*. The present honey set belonged to our previous study, where the samples were collected in 2018–2019 and their physicochemical parameters, chemical composition, mineral elements, and minimal inhibitory concentration (MIC) were screened. However, the polyphenolic profile and their antibiofilm activity on susceptible and multidrug-resistant pathogens were still unknown. According to polyphenolic profile of the honey samples, significant differences were observed according to their geographical origin in terms of the qualitative profiles. The five best honey samples (OR24.1, LR34, LO40, LO48, and LO53) belonging to *S. problanca*, *Melipona* sp., and *M. indecisa* were selected for further analysis due to their high biomass reduction values, identification of the stingless bee specimens, and previously reported physicochemical parameters. This subset of honey samples showed a range of 63–80% biofilm inhibition through biomass assays. Fluorescence microscopy (FM) analysis evidenced statistical log reduction in the cell count of honey-treated samples in all pathogens (*P* <0.05), except for *S. aureus* ATCC 25923. Concerning cell viability, *C. tropicalis*, *K. pneumoniae* ATCC 33495, and *K. pneumoniae* KPC significantly decreased (*P* <0.01) by 21.67, 25.69, and 45.62%, respectively. Finally, scanning electron microscopy (SEM) analysis demonstrated structural biofilm disruption through cell morphological parameters (such as area, size, and form). In relation to their polyphenolic profile, medioresinol was only found in the honey of Loja, while scopoletin, kaempferol, and quercetin were only identified in honey of Los Rios, and dihydrocaffeic and dihydroxyphenylacetic acids were only detected in honey of El Oro. All the five honey samples showed dihydrocoumaroylhexose, luteolin, and kaempferol rutinoside. To the authors’ best knowledge, this is the first study to analyze stingless bees honey-treated biofilms of susceptible and/or MDR strains of *S. aureus*, *K. pneumoniae*, and *Candida* species.

## Highlights

• Thirty-five samples by ten stingless bee species of Ecuador were studied.
• Biofilms of *S. aureus*, *K. pneumoniae*, *C. albicans*, and *C. tropicalis* were analyzed.
• Results showed differences in honeys' polyphenolic content between provinces.
• The five best honey samples demonstrated a range of 63–80% biofilm inhibition.

## Graphical abstract

**Figure:**
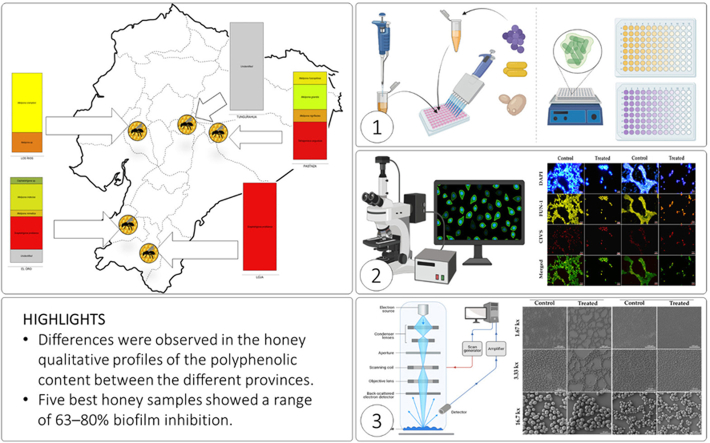

## 1 Introduction

Indigenous and rural stingless beekeepers in Ecuador hold an ancestral knowledge of applying stingless bee honey as alternative medicine in the ocular or wound healing process and against colds and throat inflammation in numerous infectious diseases (Hau-Yama et al., 2020; Rao et al., 2016; Vit et al., 2017). However, little is still known about this antimicrobial activity and its pharmaceutical applications. Ecuador is nowadays known for its megabiodiversity in stingless bees revealing a total of 132 species distributed in 23 genera found in 24 provinces. Stingless bee honeys are an essential key to discovering new antimicrobial agents from a diverse background source of different floral origins and metabolites produced by stingless bee species (Vit et al., 2018). Although its huge potential, few studies involving Ecuadorian stingless bees have been realized. The honey production of stingless bees (Tribe: *Meliponini*) is very small compared to honeybees (genus *Apis*), so its use is more directed to medicinal than food purposes (Rao et al., 2016; Vit et al., 2015; Zulkhairi Amin et al., 2018). Honey have been revealed to be potential sources of natural food preservation chemicals by preventing food spoilage through microbial planktonic and/or biofilm growth. Free radical scavenging and bleaching inhibition are essential properties of food preservatives, being honey considered as a natural presentive for food. Bioactive substances in honey include phenolic acids, vitamins, and enzymes, where honey is known for its most potent antioxidant activity due to its flavonoid concentration (Teshome et al., 2022). The antibacterial activity of stingless bee products has been widely discussed by researchers around the world (Al-Hatamleh et al., 2020; Bouchelaghem et al., 2022; Popova et al., 2021; Zulhendri et al., 2022). The intrinsic properties of honey, such as low pH, high osmolarity, low water activity, and certain antioxidant compounds (still poorly known), inhibit the growth of several microorganisms, including fungi (Proaño et al., 2021; Toaquiza Vilca, 2020). The antibacterial properties of stingless bee honey can be divided into peroxide and non-peroxide (Esa et al., 2022). Most of the antibacterial action has been attributed to hydrogen peroxide activity producing highly reactive free radicals that interact and damage pathogens (Brudzynski, 2020). The hydrogen peroxide production is slow and induces a continual antibacterial activity that could injury the host tissue if not sufficiently diluted. However, hydrogen peroxide can be quickly neutralized or blocked in the presence of catalase production (for example, in the case of *Staphylococcus* species) or other factors (such as heat) (Mama et al., 2019). Therefore, the consistent and stable antibacterial activity of stingless bee honey is usually associated with non-peroxide compounds, showing also a superior antibacterial action than *Apis mellifera* honey (Alvarez-Suarez et al., 2018; Ávila et al., 2018). The non-peroxide components of honey are derived from phytochemicals, sugar content, and acidity. The phytochemical features of honey include polyphenols and antimicrobial peptides (Alvarez-Suarez et al., 2018). Polyphenols are a heterogeneous class of chemical compounds divided into flavonoids and phenolic acids (Cianciosi et al., 2018), being in higher levels in stingless bee honey than *Apis mellifera* honey (Esa et al., 2022). The polyphenol composition in honey mainly depends on its floral origin, especially in the case of monofloral varieties (Cianciosi et al., 2018). However, little is still known about monofloral varieties in stingless bee honeys, being usually related as multifloral nature. The antimicrobial activity by polyphenols is attributed to their primary antioxidant effects, scavenging free radicals and their stabilization by releasing hydrogen from one of their hydroxyl groups and being able to inhibit virulence pathways of microbial pathogens (Alvarez-Suarez et al., 2018; Esa et al., 2022). Until now, despite the fact that some studies have tried to determine the contribution to the antimicrobial activity of several of the components of honey separately (Bucekova et al., 2018; Bugarova et al., 2021; Majtan et al., 2020), most report this activity as a consequence of the effect of the various components (peroxides and not peroxides) together. However, although it has been seen that the interaction between the bioactive compounds in a mixture enhances its biological effects, it is important to study the contribution of each one separately. Thus, future studies are necessary to detail the contribution of the main compounds, such as polyphenols, to the biological activities of honey, such as its antimicrobial activity. In fact, in stingless bee honey, the flavonoid compounds reported are naringenin, kaempferol, apigenin, pinocembrin, and chrysin; while phenolic acids are protocatequic acid, *p-* hydroxibenzoic acid, caffeic acid, chlorogenic acid, vanillic acid, *p*-coumaric acid, benzoic acid, ellagic acid, and cinnamic acid (Ng et al., 2020; Esa et al., 2022), many of which have been associated with important antimicrobial activities (Manso et al., 2022). Although antimicrobial peptides were found in stingless bee honey (Nishio et al., 2016), Ng and colleagues considered phenolic and flavonoid compounds to be the only phytochemicals that contributed to the non-peroxide antibacterial activity of stingless bee honey (Esa et al., 2022; Ng et al., 2020). For this reason, the present study aims to evaluate the polyphenolic composition in Ecuadorian stingless bee honey due to their megabiodiversity but relatively few studies on the stingless bees (Villacrés-Granda et al., 2021; Vit et al., 2018).

Recent studies have shown that stingless bee honey exhibits a stronger antimicrobial effect compared to honey from domestic bees even in multidrug-resistant (MDR) bacteria strains (Kot et al., 2020; Villacrés-Granda et al., 2021), as well as several fungal species of clinical interest (Fonte-Carballo et al., 2016; Hau-Yama et al., 2020; Manrique and Santana, 2008) and agricultural importance (Albores-Flores et al., 2018). However, little is known about its impact on biofilms, which are frequently associated with more virulent infections and are resistant to conventional therapies, especially in patients with chronic wounds, prostheses, burns, or diabetes (Pinto et al., 2021). Nowadays, more and more research is being carried out regarding antibiofilm agents that can act alone or in synergy and stingless bee honeys have been considered an important source of bioactive compounds with relevant biological properties (Alvarez-Suarez et al., 2018; Biluca et al., 2016). However, the mechanisms involved remain unknown, and only a few possible related bioactive compounds have been described in the literature (Ávila et al., 2018; Brudzynski, 2021; Proaño et al., 2021). Some examples of known antibacterial compounds are p-coumaric acid, quercetin, and hesperetin previously identified in Brazilian stingless bee honey by Ávila and colleagues (Ávila et al., 2019a) with antibiofilm activity against two MDR human pathogens *Staphylococcus aureus* MRSA S21 and *Pseudomonas aeruginosa* P28. Vitamin C and several organic acids (such as oxalic acid, lactic acid, acetic acid, and citric acid) were also identified in Ecuador stingless bee honey inhibiting microbial growth in gram-negative and gram-positive MDR human pathogens (Villacrés-Granda et al., 2021), which the same honey sample set had been selected for further evaluation in the present study. Against this background, the aims of this research were to characterize polyphenolic composition and evaluate the antibiofilm activity of native stingless bee honey samples from five different provinces of Ecuador (Tungurahua, Pastaza, El Oro, Los Ríos, and Loja) against MDR pathogens associated with wound healing and mucosal epithelia infections, more exactly, *Staphylococcus aureus*, *Klebsiella pneumoniae*, *Candida albicans*, and *Candida tropicalis*. The antibiofilm activity was analyzed through biomass assays, fluorescence (total cell count and cell viability), and scanning electron (structural biofilm composition) microscopy.

## 2 Materials and methods

### 2.1 Isolates and growth conditions

Two bacterial and two fungal species were selected for the present study. For bacterial species, *Staphylococcus aureus* and *Klebsiella pneumoniae* were chosen as representative examples of well-known gram-positive and -negative pathogens, respectively (Tacconelli et al., 2018). For each bacterial species, one MDR strain and one susceptible strain were used in this study. The two MDR strains were *Staphylococcus aureus* MRSA 333 and *Klebsiella pneumoniae* KPC 609803, while the two susceptible strains were *Staphylococcus aureus* ATCC 25923 and *K. pneumoniae* ATCC 33495. In previous studies, *Staphylococcus aureus* MRSA 333 was isolated from nasal and pharyngeal volunteers from Universidad de Las Americas in Quito (Ecuador) (Proaño et al., 2021; Villacrés-Granda et al., 2021), and *Klebsiella pneumoniae* KPC 609803 was donated from the collection of clinical isolates at Zurita & Zurita Clinical Laboratories (http://www.zuritalaboratorios.com) in Quito (Ecuador) (García-Tenesaca et al., 2018). As previously described, *S. aureus* MRSA 333 is resistant to penicillin and oxacillin (Bastidas et al., 2019) and *K. pneumoniae* KPC 609803 is resistant to imipenem and ertapenem (García-Tenesaca et al., 2018); however, their resistance profiles were confirmed for this study through antibiograms. In addition, *Candida albicans* and *Candida tropicalis* were elected as representative examples of well-known *Candida* species associated with opportunistic infections (Atiencia-Carrera et al., 2022a, 2022b), more precisely *C. albicans* ATCC 1023 and *C. tropicalis* isolates from the microbial collection of the Institute of Microbiology, Universidad San Francisco de Quito (designated as IMUSFQ-V546). *C. tropicalis* isolate IMUSFQ-V546 was previously recovered from a patient with invasive candidiasis and identified through DNA sequences at multiple loci and biochemical properties at the National Institute for Research in Public Health (INSPI). Before biofilm assays, each microorganism was previously cultured in Tryptic Soy Broth (TSB) for 24 h at 37 °C and then microbial growth was adjusted to 0.5 McFarland with phosphate-buffered saline (PBS) to obtain an estimated cellular density of 1.5 x10^8^ colony-forming units (CFU)/mL for bacterial strains, 1.5x10^6^ CFU/mL for *C. albicans*, and 3.0 x10^6^ CFU/mL for *C. tropicalis* (Guinea et al., 2010).

### 2.2 Honey samples

The study set included thirty-five different honey samples produced by ten different stingless bee species (*Cephalotrigona* sp., *Melipona* sp., *M. cramptoni*, *M. fuscopilosa*, *M. grandis*, *M. indecisa*, *M. mimetica*, *M. nigrifacies*, *Scaptotrigona problanca*, and *Tetragonisca angustula*) from five provinces of Ecuador, namely Tungurahua, Pastaza, El Oro, Los Ríos, and Loja. These stingless bee honey samples were collected during 2018 and 2019 by Villacrés-Granda et al. (2021) directly from the artisanal hives of stingless beekeepers in the general pot honey production zones. The samples belonged from three geographical regions of mainland Ecuador: the Andean (7 samples from Tungurahua and Loja provinces with *S. problanca*), Amazonian (9 samples from Pastaza province with *M. fuscopilosa*, *M. grandis*, *M. nigrifacies*, and *T. angustula*) and Coastal Pacific (19 samples from El Oro and Los Ríos provinces with *Cephalotrigona* sp., *Melipona* sp., *M. cramptoni*, *M. indecisa*, *M. mimetica*, and *S. problanca*) regions during both dry and rainy seasons (see Supplementary Table S1). Samples were donated by stingless bee beekeepers registered at the Ecuadorian Agency for Agricultural Quality Assurance (AGROCALIDAD, Ecuador). The honey samples were collected in sterilized plastic containers and stored at 4–6 °C in the dark until further preparation. The identification of the stingless bee specimens, as well as the physicochemical characterization of honey samples, was determined beforehand as previously reported (Villacrés-Granda et al., 2021).

For the anti-microbiological capacity studies developed here, the collected samples were first centrifuged at 2500 rpm for 10 min and the supernatants were then filtered through a 0.22-μm filter unit to avoid the development of microorganisms typical of honey (de Sousa, 2021; Echeverrigaray et al., 2021; Rosa et al., 2003; Silva et al., 2017). Stock solutions of each sample were prepared at 50% (v/v) diluted in PBS. For all experimental assays, the honey concentration was set up to 15% (v/v), at which point previous studies showed significant antimicrobial activity among honey samples through minimal inhibitory concentration (MIC) and biofilm inhibition and eradication assays (Proaño et al., 2021; Villacrés-Granda et al., 2021). In addition, a solution of artificial honey lacking H_2_O_2_ was made as osmotic control, which is a normal product of glucose oxidation consisting of 1.5 g sucrose, 7.5 g maltose, 40.5 g fructose, and 33.5 g glucose in 17 mL of deionized water. This osmotic control aimed to evaluate the contribution of the predominant honey sugars to the biofilm inhibition assays and was also evaluated at 15% (v/v) diluted in PBS (Cooper et al., 2002).

### 2.3 HPLC/ESI/MS-MS analysis of polyphenolic composition of honey

For the extraction of phenolic compounds, a portion of each honey was dissolved in acidified water (pH 2 with HCl) and deposited in a Sep-Pak® C18 Plus Short SPE Cartridge (Waters Co., Milford, MA) previously activated with 10 mL of methanol followed by 10 mL of water. Next, the cartridge was washed with 100 mL of water in order to eliminate the sugars and other retained substances. Finally, the phenolic compounds were eluted with 3 mL of methanol and dried using a Büchi® Rotavapor® R-210 (Merck KGaA, Darmstadt, Germany) set at 40 °C of the water bath and the cooling water at 4 °C until the solvent was eliminated (Truchado et al., 2008). Phenolic compounds were identified and quantified by HPLC-DAD Mass Spectrometry (MS/MS) using an Agilent Technologies” 1200 Series Binary SL (Agilent Technologies, Palo Alto, CA, USA). The separation was carried out in a Poroshell 120 EC-C18 column, with dimensions 2.7 μm, 150 mm × 4.6 mm, at a temperature of 35 °C. As mobile phase, 0.1% formic acid (eluent A) and acetronitrile (ACN) (eluent B) have been used at a flow rate of 0.5 ml/min. The gradient program was as follows: 15% of B over 5 min, from 15 to 20% over 5 min, from 20 to 35% over 10 min, from 35 to 50% B over 10 min, from 50 to 60% over 2 min, isocratic by 5 min and finally from 60 to 80% over 3 min. The HPLC-DAD system was connected in parallel with an Applied Biosystems 3200 QTRAP® LC/MS/MS System (MDS Sciex, Darmstadt, Germany) mass spectrometer (MS). Then, 330 and 360 nm were selected as preferred wavelengths for the DAD and in the MS, operated in the negative ion mode, at a temperature of 400 °C and recording the spectra between *m/z* 100 and *m/z* 1500. Zero air was used as nebulizing gas (30 psi) and turbo gas (400 °C, 40 psi) for drying the eluent, and nitrogen as curtain gas (20 psi) and medium collision gas. The detection method used was full scan at high sensitivity (Enhanced MS, EMS) with the following parameters: capillary voltage, - 4500 V with the following potentials: decluttering potential (DP) −50 V, input potential (EP) - 6 V, and collision energy (CE) −10 V. In parallel to this analysis, an Enhanced Product Ion (EPI) mode was carried out, to obtain the characteristic fragmentation pattern of the majority ion obtained in the first experiment. In this case, the conditions used were DP -50 V, EP -6 V, CE -25 V and collision energy spread (CES) 0 V. The identification of the phenolic compounds was carried out based on the UV–Visible spectra, and the MS and MS^2^ data obtained from the mass spectrometer. Compounds were identified by their retention time, UV–Vis spectra and mass spectra. UV–Vis spectra were compared with our data library (in-house library) and standards when available (see Supplementary Fig. S1). On the other hand, mass spectra were compared with those described in the literature and with the predicted MS/MS spectra available in the fooddb database (https://foodb.ca) (see Supplementary Table S2).

Total polyphenolic content was determined throughout the Folin-Ciocalteu method (Singleton et al., 1999). A calibration curve was obtained using an external standard for gallic acid (0.19–1 mM; y = 1.7443x − 0.1444, R^2^ = 0.99), and the results were expressed as mg of gallic acid equivalents (GAE) per 100 g of honey (mg GAE/100 g) (see Supplementary Table S3).

### 2.4 Biofilm inhibition and eradication assays

The inhibition and eradication of biofilms by honey samples in the present study were evaluated by biofilm biomass quantification. Further analysis was realized by evaluating the biofilm inhibition assays through the total cell count together with cell viability and the structural biofilm composition. The biofilm biomass quantification was realized through an optical density (OD) assay with crystal violet (CV) staining, as previously reported (Gulati et al., 2018). The total cell count and cell viability analysis were performed through fluorescence microscopy (FM) using DAPI and LIVE/DEAD assays. Finally, structural biofilm composition was evaluated through scanning electron microscopy (SEM) analysis. Each type of biofilm inhibition assay was performed with at least three replicates per microorganism on different days and, in each replicate assay, two samples of biofilm by microorganism were analyzed.

#### 2.4.1 Optical density assay with crystal violet staining

As previously described, fresh growth cultures of each microorganism were adjusted to 0.5 McFarland with PBS before the preparation of 96-well plates (Guinea et al., 2010). The biofilm inhibition and eradication assays were carried out according to a previous study with slight modifications (Sornsenee et al., 2021). To the 96-well plates for biofilm inhibition, 110 μL of TSB, 100 μL of inoculum 0.5 McFarland, and 90 μL of the honey sample stock solution were added. In addition, positive and negative controls were added in each assay. Positive controls consisted of wells with 110 μL TSB, 100 μL of inoculum, and 90 μL PBS, while negative controls were 110 μL of TSB and 190 μL of PBS. After the initial preparation, the 96-well plate was incubated for 24 h at 37 °C under a constant orbital agitation of 120 rpm. To evaluate the honey samples’ ability to inhibit biofilm biomass formation, we used an optical density assay with crystal violet (CV) staining using a modified version of the method suggested by Peeters et al. (2008). Briefly, the fixation step was realized with 200 μL of methanol 100% (v/v) for 20 min, and the biofilms were stained with 200 μL of CV solution at 1% (v/v; Merck, Darmstadt, Germany) for 20 min. Each well was washed twice with 200 μL of PBS and then decolored with 200 μL of glacial acetic acid at 99.8% (v/v; ThermoFisher Scientific, Massachusetts, USA). Finally, the optical density at 630 nm (OD 630 nm) of the 96-well plate was read in the spectrophotometer ELISA Elx808 (BioTek, Winooski, USA), removing the absorbance values of the negative controls from the remaining wells and considering positive controls as the total biofilm formation for each microorganism. After the initial evaluation of the potential antibiofilm activity by stingless bee species, the honey samples with the highest inhibition rates on biofilm biomass in each microorganism were selected for further FM and SEM analysis. For biofilm eradication evaluation, similar procedures and controls were realized apart, from previous 24h-biofilm samples grown under the same experimental conditions, followed by washing steps and then the honey samples were added to the fresh medium at 15% (v/v) in the wells. An additional incubation of 24 h under the same conditions was realized before the 96-well plate was washed and then read in the spectrophotometer at OD630 nm.

#### 2.4.2 Fluorescence microscopy analysis

The total cell count and cell viability evaluation through FM analysis were performed in 6-well plates containing a sterile coverslip as an abiotic surface for biofilm development (Chandra and Mukherjee, 2015). Each 6-well plate contained honey-treated samples, negative controls, or positive controls. For positive controls, duplicate wells were filled with 100 μL of appropriate microbial inoculum in PBS solution and 2.9 mL of sterile TSB. For negative controls, duplicate wells were set up with 100 μL of PBS and 2.9 mL of sterile TSB. Lastly, honey-treated wells were set up with 100 μL of appropriate microbial inoculum and 2.9 mL of TSB containing 15% (v/v) of the selected honey samples (from previous stock solutions) or a solution of artificial honey (osmotic control). Then, the 6-well plates were incubated for 24 h at 37 °C with a constant orbital agitation of 120 rpm. After the realization of 24h-biofilm assays, the medium was carefully removed from the wells and the coverslips were also carefully washed with 3 mL of sterile PBS to remove the growth medium and the planktonic cells. Each coverslip containing the biofilm sample was then placed in a sterile plastic flask with 3 mL of sterile PBS, scrapped, and vortexed at maximum velocity for 5 min to ensure that the biofilm was removed from the coverslip and entered the PBS solution, as described in the literature (Castro et al., 2022). Finally, the total cell count and cell viability evaluation through FM analysis were realized using 200 μL of the PBS solution containing biofilm cells in a new and sterile coverslip.

For total cell count evaluation, DAPI (4',6-diamidino-2-phenylindole, dihydrochloride; D3571, ThermoFisher Scientific, Massachusetts, USA) fluorescence staining was used in the recovered coverslips by applying a working solution of 300 nM in PBS. A further analysis was done through DAPI staining to study the structure and composition of biofilm bridges and extracellular DNA (eDNA), as previously described in other studies (Ducret et al., 2016; Lei et al., 2019; Zatorska et al., 2017). The total cell count by DAPI staining was also used to optimize the cell viability assays of all bacterial and yeast-related biofilms using two different LIVE/DEAD kits, as previously described (Atiencia-Carrera et al., 2022b). The two different LIVE/DEAD kits to evaluate the cell viability were the LIVE/DEAD Yeast Viability Kit (L7009, ThermoFisher Scientific, Massachusetts, USA) for *Candida* species and LIVE/DEAD BacLight Bacterial Viability Kit (L7012, ThermoFisher Scientific, Massachusetts, USA) for the remaining microorganisms. Working solutions were prepared according to the manufacturer's manuals and stored at −20 °C. Briefly, the final concentrations for the bacterial assays were 6 μM of Syto-9 and 30 μM of propidium iodide, and the final concentrations for the fungal assays were 10 μM of FUN-1 and 25 μM of calcofluor white M2R. After the fixation of the previous 200 μL PBS solution containing biofilm cells in a sterile coverslip by drying at room temperature or incubation (at 60 °C), 200 μL of LIVE/DEAD working solution was carefully applied to treated and untreated biofilm samples and incubated at room temperature in the dark for 15 min. The samples were washed twice with 200 μL of PBS to remove excess fluorescent dyes and 200 μL of DAPI working solution was added to the samples, making sure that the biofilms were completely covered. The coverslips were further incubated at room temperature in the dark for 10 min. Finally, the samples were washed twice with PBS to remove the excess DAPI and were dried at room temperature (25 °C) in the dark until EM analysis. Images were obtained with an Olympus BX50 microscope equipped with the MU633-FL digital camera (AmScope, AmScope, California, USA) and digitized with AmScope version 1.2.2.10. As previously described (Rosenberg et al., 2019), 15 images per sample were taken following a zigzag pattern from top to bottom trying to cover the entire surface of the coverslip. For more reproducible results presentation, the counted cells were given per frame (9600 μm^2^) of the visual observation at 1000x on the fluorescence microscope. Images were merged in Fiji-ImageJ version 1.57 (Schindelin et al., 2012). The Fiji-ImageJ software was also used to obtain total cell counts and content of extracellular polymeric substance (EPS) in DAPI images (Lippi et al., 2019). For cell viability, the percentages of dead and alive cells within images were measured using Fiji-ImageJ software, specifically the macros Biofilms Viability checker proposed by the plugin MorphoLibJ (Legland et al., 2016; Mountcastle et al., 2021).

#### 2.4.3 Scanning electron microscopy analysis

For SEM analysis, sterile 22-mm circular cover glasses (Heathrow Scientific, Vernon Hills, Illinois, USA) were placed in 6-well plates and 24h-biofilm assays were realized as previously described in EM analysis. Wells containing biofilm samples were fixed with a solution of 4% glutaraldehyde in PBS with adjusted pH (similar to growth broth) for 1 h. A post-fixation step was carried out with 1% osmium tetroxide in cacodylate buffer for 1 h and then samples were treated with 1% tannic acid for an additional 1 h. The samples were dehydrated through a series of immersion steps with different ethanol solutions (30%, 50%, 70%, 80%, 90%, and 100% at HPLC grade; v/v in distilled water) for 30 min for each one. The samples were subsequently frozen with liquid nitrogen and dried for 4 days in a freeze dryer (−50 °C, 0.400 hPa). Finally, cover glasses containing biofilm samples were coated with gold through a sputter coating machine (Quorum, Q150R ES, UK) and SEM analysis was performed using a Tescan Mira 3 scanning electron microscope equipped with a Schottky Field Emission Gun (Schottky FEG-SEM, MIRA III TESCAN, Brno, Czech Republic) at the Centro de Nanociencia y Nanotecnología of the Universidad de las Fuerzas Armadas (ESPE), as previously described (Pilaquinga et al., 2019). Morphology parameters and shape descriptors of the cells were also obtained from all images via Fiji-ImageJ version 1.57 (Schindelin et al., 2012), as reported in other studies (Ducret et al., 2016; Lobo et al., 2016; Prodanov and Verstreke, 2012). At least 150 cells were sampled for comparison between untreated and treated-biofilm samples through different morphological parameters and shape descriptors, as reported by others (Casar et al., 2021; Lobo et al., 2016; Sieniawska et al., 2015; Sridhar et al., 2021). Finally, fractal dimension was estimated from the slopes of cross-correlation functions to describe biofilm morphology, as previously described in other studies (Beyenal et al., 2004; Hermanowicz et al., 1995, 1996; Lewandowski et al., 1999; Picioreanu et al., 1998).

### 2.5 Statistical analysis

For pairwise comparison between control and honey-treated samples, the Wilcoxon nonparametric test was applied, except for the fractal dimension analysis, where due to sample size, the *t*-test was applied. In the preliminary antibiofilm activity among the honey set screening through OD assays, the Wilcoxon test with Holm–Bonferroni adjustment for multiple comparisons was realized to detect differences between stingless bee species and osmotic controls. All data analysis was carried out through R studio software version 4.0 (RStudio Team, 2015) using several R packages: “ggpubr”, “ggplot2”, “gapminder”, “Rmisc”, “rstatix”, “forcats”, and “tidyverse” (Bryan, 2017; Hope, 2022; Kassambara, 2020, 2021; Wickham, 2016; Wickham et al., 2019). All *P*-values below or equal to 0.05 were considered statistically significant.

## 3 Results

### 3.1 Analysis of polyphenolic composition of honey samples

Table 1 shows a comparison of the different groups of compounds identified in the honey samples from the five provinces. Notable differences were observed in the honey qualitative composition when compared between the different provinces, in particular in the presence of certain phenolic acids and flavonoids. Despite the variety of bee species among honey samples of certain province (e.g., El Oro province), the qualitative profile showed a very similar honey composition suggesting a strong correlation between the type of polyphenols in honey samples and their floral origin background. A similar behavior was observed in the total content of polyphenols (Supplementary Table S3). In this case, the Folin-Ciocalteu method was used. According to the data obtained, the samples from the province of Loja presented higher polyphenolic content with values that ranged between 109.44 and 171.96 mg GAE/100 g of honey. Although this method is less precise than HPLC, since it evaluates the presence of reducing substances as a whole, it is accepted by the scientific community as an index of the total content of polyphenols, which allows comparison with a larger group of available data on the content of these compounds. Taking these data into account, and those obtained from the HPLC result, Supplementary Fig. S1 shows a representative chromatogram (recorded at 360 nm) where the chromatograms obtained from honey samples from Los Ríos (LR), Loja (LO) and El Oro (OR) (see Supplementary Fig. S1). In this chromatogram it can be seen that the complexity of the profile, as well as the absorbance -proportional to the content of phenolic compounds- are consistent with the GAE values shown in Supplementary Table S3.

**Table 1:** Comparison of the different groups of compounds identified in the honey samples of different species of stingless bee honey from the Loja, Los Ríos, and El Oro provinces from Ecuador.

Phenolic family	Tentatively identified compound	Samples				
		Loja	Los Rios	El Oro	Tungurahua	Pastaza
Lignans	Medioresinol	X				
Coumarins	Scopoletin-hexose-pentose derivative		X			
	Scopoletin		X		X	
	Coumarin (e.g. dicoumarin) glycoside		X			
	Aesculin		X			
	Psoralen derivative		X			
Phenolic acids	Caffeic acid	X				
	Dihydrocaffeic acid			X		
	Caffeoyl dihexose		X			
	Caffeic acid cinnamyl ester		X			
	Feruloylhexose					X
	Feruloylcaffeoylquinic acid				X	
	Dihydrocoumaroylhexose	X	X	X		
	Sinapic acid					X
	Hydroxybenzoic acid					X
	Protocatechuic acid					X
	Syringic acid					X
	Dihydroxyphenylacetic acid			X		
	Hydroxyphenylacetic acid	X				X
	Phenyllactic acid		X			
	Abscisic acid	X			X	
	Carnosic acid	X				
	Rosmarinic acid derivative					X
**Flavonoids**						
Flavones	Luteolin	X	X	X		X
	Chrysoeriol	X				
	Methoxyflavone	X				
Flavanones	Hesperetin hexoside	X				
	Sakuranetin glycoside		X			
	Sakuranetin	X				
	Pinobanksin		X			
	Eriodictyol		X			X
Flavonols	Kaempferol deoxyhexoside glucuronide			X		
	Kaempferol sophoroside rhamoside	X				
	Kaempferol hexoside rutinoside			X		
	Kaempferol neohesperidoside rhamnoside	X				
	Kaempferol neohesperidoside	X				
	Kaempferol rutinoside	X	X	X	X	
	Kaempferol hexoside pentoside	X				
	Kaempferol hexoside		X			
	Kaempferol rhamnoside			X		
	Methylkaempferol rutinoside pentoside			X		
	Methylkaempferol pentoside			X		
	Kaempferol	X		X		X
	Quercetin rutinoside	X	X			
	Quercetin rhamnoside	X	X			X
	Quercetin hexoside		X			
	Hydroxyethylquercetin	X				
	Quercetin	X	X	X		X
	Isorhamnetin rutinoside hexoside			X		
	Isorhamnetin rutinoside	X				X
	Isorhamnetin hexosyl(1→2)hexoside		X			
	Isorhamnetin glucuronide		X	X		
	Isorhamnetin hexoside		X			
	Isorhamnetin		X	X		
	Tamarixetin		X			
	Dimethylquercetin hexoside		X			
	Dimethylquercetin		X			
Chalcone	Phlorizin		X			
Isoflavones	Pseudobaptigenin	X				
Others	Lumicrome			X		

Honey samples from the province of Loja were the only ones in which lignan were identified (medioresinol), while they also showed a wide variety of compounds from the families of phenolic acids (*e.g.*, caffeic, hydroxyphenylacetic, abscisic and carnosic acid), flavones (*e.g.*, luteolin and chrysoeriol), flavanones (*e.g.*, hesperetin and sakuranetin), as well as flavonols, of which this type of honey showed the greatest variety, mainly glycosides of kaempferol and quercetin. On the other hand, the honeys from the province of Los Rios showed an interesting profile since they were the only ones to exhibit several types of coumarins (*e.g.*, scopoletin-hexose-pentose derivate, scopoletin, coumarin glycoside, aesculin, and psoralen), except for scopoletin in some honeys from Tungurahua. These Los Ríos honey samples also specifically showed the presence of certain phenolic acids (*e.g.*, caffeoyl dihexose, caffeic acid cinnamyl ester, and phenyllactic acid) and a wide and varied content of flavonoids from different families such as flavanones (*e.g.*, sakuranetin glycoside, pinobanksin, and eriodictyol) and flavonols (*e.g.*, kaempferol hexoside, quercetin hexoside, isorhamnetin hexosyl, isorhamnetin hexoside, tamarixetin, and dimethylquercetin), as well as phlorizin from the chalcone group of compounds. However, luteolin of flavones was detected in all provinces honey samples excepting for Tungurahua samples, while kaempferol rutinoside of flavonols was identified in all provinces honeys samples excepting for Pastaza samples.. Meanwhile, the El Oro honey samples showed a richer profile in compounds from the flavonoid group, mainly from the flavonol family, among which the glycosides of kaempferol and quercetin stood out. The El Oro and Loja honey samples were the only province groups to evidence the presence of lumicrome and pseudobaptigenin compounds, respectively. However, although in smaller quantity and variety, a compound from the family of flavones (luteolin) and phenolic acids (*e.g.*, dihydrocaffeic, dihydroxyphenylacetic, and dihydrocoumaroyl glucose) were also identified in El Oro honey samples. In contrast, honeys from Tungurahua showed a simpler profile with only the presence of kaempferol rutinoside together with abscisic and feruloylcaffeoylquinic acids, while samples from Pastaza presented a complex profile with numerous phenolic acids, both hidroxicinnamic and hidroxibenzoic derivatives, and different flavonoids (luteolin, eriodictyol, kaempferol and quercetin derivatives).

### 3.2 Initial screening of the honey sample set

The initial sample set revealed a diversity of the antibiofilm activity by stingless bee species in biomass reduction of different pathogens when compared to positive and osmotic controls, *i.e.*, untreated biofilm samples and treated-biofilm samples with artificial honey lacking H_2_O_2_ and other bioactive compounds (only with sugar products of glucose oxidation), respectively. As shown in Table 2, we initially analyzed the biomass reduction average produced by all honey samples per stingless bee species independently of their province (or floral) origin and statistically compared against the osmotic controls (artificial honey with sucrose, maltose, fructose, and glucose). This initial analysis of the data set allowed us to evaluate the presence of specific bee-associated species features with antimicrobial properties and differentiate biomass reduction induced by the osmotic pressure of the sugars normally present in honey samples (osmotic controls). No statistical differences were observed between the mean results of a particular stingless bee species in 24-h treated biofilms and their osmotic controls among the selected bacterial pathogens. This lack of statistical significance among bacteria suggests the absence of specific bee-associated species features with antimicrobial properties and supports the importance of certain compounds related to the floral background. However, *Candida albicans* showed statistical differences among several stingless bee species (mainly *Melipona* species) versus the osmotic control, which demonstrated the ability of *Candida albicans* to use the honey-associated sugars for its growth and not be affected by the osmotic pressure normally present in honey samples due to the biomass growth of 135.94% in the osmotic control when compared to the positive control. Interestingly, *Candida tropicalis* showed a statistical biomass reduction when treated with honey samples from *Cephalotrigona* sp., suggesting the presence of *Cephalotrigona*-associated compound(s) with biological properties against this species. Therefore, further analysis into biomass reduction was realized throughout the honey sample set from every stingless bee species, selecting the most promising honey samples for each microbial pathogen of the present study (see Supplementary Materials). As shown in Table 3, the five best honey samples (OR24.1, LR34, LO40, LO48, and LO53) were selected due to their high biomass reduction values (63–80% of biofilm inhibition), identification of the stingless bee specimens in honey samples (*Melipona* sp., *Melipona indecisa*, and *Scaptotrigona problanca*), and previously reported physicochemical parameters of the honey samples by our research group (Villacrés-Granda et al., 2021). These honey samples showed the highest values of biofilm reduction in inhibition assays out of the six microorganisms, belonging to three specific provinces of Ecuador (Loja, El Oro, and Los Rios) and two stingless bee genera (*Scaptotrigona* and *Melipona* spp.). Furthermore, these honey samples showed statistically significant values in the inhibition of biofilms among pathogens (*P* < 0.01) when compared to the osmotic controls, except for *Staphylococcus aureus* strains. When exposed to honey samples, the biofilm formation was between 19.96 and 36.80%, with *Candida albicans* ATCC 10231 being the most inhibited pathogen from our group set. The *Melipona indecisa* species OR24.1 sample demonstrated the highest inhibition and statistical difference value against *Klebsiella pneumoniae* KPC 609803, showing 36.80% in biofilm formation when compared to its osmotic control (76.94%). Likewise, a preliminary analysis was realized with the same honey samples through biofilm eradication assays to evaluate the disruption of pre-established biofilms. The eradication of established 24-h biofilms showed less efficiency, evidencing biofilm rates of 43.90–118.39%, where treated-biofilm samples of *Staphylococcus aureus* strains and *Klebsiella pneumoniae* ATCC 33495 showed an increment in biofilm formation (see Table 3). From the initial assessment of our honey sample set on biofilm biomass reduction by optical density assays, we further evaluated the potential antibiofilm activity of the best honey samples on the biofilm inhibition assays through fluorescence microscopy (FM) and scanning electron microscopy (SEM) analysis.

**Table 2:** Initial evaluation of the potential antibiofilm activity by stingless bee species in biomass reduction through biofilm inhibition assays of different pathogens when compared to positive and osmotic controls.

Stingless bee species and controls	*S. aureus*				*K. pneumoniae*				*C. albicans*		*C. tropicalis*	
	ATCC 25923		MRSA 333		ATCC 33495		KPC 609803		ATCC 10231		V546	
	A_630_	Biomass^1^,%	A_630_	Biomass^1^,%	A_630_	Biomass^1^,%	A_630_	Biomass^1^,%	A_630_	Biomass^1^,%	A_630_	Biomass^1^,%
***Cephalotrigona* sp.**	0.169 (0.126)	38.54 (28.71)	0.145 (0.081)	40.38 (22.42)	0.226 (0.017)	55.29 (4.21)	0.239 (0.013)	174.16 (9.24)	**0.086 (0.022)**	**42.53 (10.70)**	**0.044 (0.011)**	**17.49 (4.26)**
***Melipona cramptoni***	0.162 (0.073)	36.93 (16.73)	0.112 (0.048)	31.07 (13.28)	0.378 (0.106)	92.35 (25.87)	0.122 (0.049)	89.32 (35.88)	**0.062 (0.018)**	**30.74 (8.83)**	0.144 (0.063)	56.76 (24.71)
***Melipona fuscopilosa***	0.202 (0.017)	46.14 (3.84)	0.224 (0.019)	62.22 (5.20)	0.534 (0.050)	130.63 (12.21)	0.230 (0.057)	167.97 (41.74)	**0.160 (0.007)**	**78.99 (3.54)**	0.326 (0.231)	128.66 (90.97)
***Melipona grandis***	0.191 (0.047)	43.60 (10.78)	0.250 (0.068)	69.31 (18.74)	0.428 (0.052)	104.57 (12.71)	0.208 (0.062)	151.54 (45.09)	**0.146 (0.084)**	**71.86 (41.12)**	0.220 (0.068)	86.83 (26.69)
***Melipona indecisa***	0.153 (0.048)	35.09 (10.93)	0.211 (0.051)	58.70 (14.02)	0.283 (0.055)	69.16 (13.52)	0.095 (0.042)	69.32 (30.76)	**0.110 (0.020)**	**54.47 (9.68)**	0.173 (0.053)	68.45 (20.84)
***Melipona mimetica***	0.112 (0.021)	25.59 (4.74)	0.147 (0.009)	40.78 (2.43)	0.298 (0.021)	72.82 (5.05)	0.191 (0.034)	139.36 (24.63)	**0.110 (0.006)**	**54.34 (2.75)**	0.189 (0.055)	74.79 (21.83)
***Melipona nigrifacies***	0.154 (0.035)	35.12 (12.75)	0.173 (0.043)	48.14 (11.78)	0.390 (0.059)	95.45 (14.47)	0.159 (0.035)	116.41 (25.68)	**0.113 (0.020)**	**55.89 (9.73)**	0.288 (0.115)	113.41 (45.35)
***Melipona* sp.**	0.154 (0.035)	35.23 (7.87)	0.121 (0.043)	33.64 (11.94)	0.214 (0.079)	52.43 (19.28)	0.069 (0.012)	50.51 (8.97)	**0.149 (0.011)**	**73.61 (5.38)**	0.188 (0.069)	74.27 (27.33)
***Scaptotrigona problanca***	0.161 (0.076)	36.88 (17.43)	0.150 (0.062)	41.73 (17.20)	0.240 (0.108)	58.67 (26.38)	0.127 (0.043)	92.46 (31.34)	**0.099 (0.040)**	**48.95 (19.46)**	0.131 (0.081)	51.68 (31.98)
***Tetragonisca angustula***	0.140 (0.038)	31.92 (8.59)	0.142 (0.080)	39.35 (22.10)	0.197 (0.099)	48.35 (24.18)	0.091 (0.039)	66.30 (28.52)	**0.077 (0.030)**	**38.20 (14.53)**	0.166 (0.121)	65.63 (47.67)
**Osmotic control**	0.112 (0.046)	25.53 (10.48)	0.101 (0.043)	28.08 (11.95)	0.218 (0.034)	53.42 (8.21)	0.105 (0.049)	76.94 (35.82)	0.276 (0.039)	135.94 (19.35)	0.141 (0.030)	55.69 (11.77)
**Positive control**	0.438 (0.123)	100.00 (28.05)	0.360 (0.070)	100.00 (19.49)	0.409 (0.062)	100.00 (15.06)	0.137 (0.035)	100.00 (25.66)	0.203 (0.048)	100.00 (23.63)	0.253 (0.047)	100.00 (18.40)

**Legend:** The table illustrates the average results of the optical density at 630 nm (A_630_) and the biofilm biomass percentages (Biomass) with their standard deviation values (SD). All assays were realized in triplicate on different days. ^1^Biomass relative to the treated-biofilm sample when compared to the positive control (untreated biofilm assays). Bold values illustrate a significative difference between the treated-biofilm sample and the osmotic control through the Wilcoxon test with Holm–Bonferroni adjustment for multiple comparisons, showing *P*-values *<*0.05.

**Table 3:** Summary of the highest biomass reduction of specific honey samples from the selected stingless bee species through biofilm inhibition and eradication assays in each pathogen and their statistical comparison with the osmotic control.

Biofilm inhibition assays							
Microorganism	Strain	Honey sample	Province of origin	Stingless bee specie	A_630_	Biomass in %	Pairwise comparison^1^, *P*-values
*Candida albicans*	ATCC 10231	LO40	Loja	*Scaptotrigona problanca*	0.040 (0.016)	19.96 (8.08)	0.000143
*Candida tropicalis*	V546	LO53	Loja	*Scaptotrigona problanca*	0.070 (0.011)	27.74 (4.56)	0.000018
*Staphylococcus aureus*	ATCC 25923	OR24.1	El Oro	*Melipona indecisa*	0.100 (0.014)	22.95 (3.26)	0.879
	MRSA 333	LR34	Los Rios	*Melipona* sp.	0.097 (0.037)	26.99 (10.17)	0.858
*Klebsiella pneumoniae*	ATCC 33495	LO48	Loja	*Scaptotrigona problanca*	0.0905 (0.011)	23.32 (2.65)	0.00000000117
	KPC 609803	OR24.1	El Oro	*Melipona indecisa*	0.050 (0.012)	36.80 (8.94)	0.002

Biofilm eradication assays							
Microorganism	Strain	Honey sample	Province of origin	Stingless bee specie	A_630_	Biomass in %	Pairwise comparison^1^, *P*-values
*Candida albicans*	ATCC 10231	LO40	Loja	*Scaptotrigona problanca*	0.064 (0.013)	61.00 (12.24)	0.0000000573
*Candida tropicalis*	V546	LO53	Loja	*Scaptotrigona problanca*	0.020 (0.009)	43.90 (18.42)	0.0110
*Staphylococcus aureus*	ATCC 25923	OR24.1	El Oro	*Melipona indecisa*	0.398 (0.017)	103.99 (4.51)	0.100
	MRSA 333	LR34	Los Rios	*Melipona* sp.	0.387 (0.019)	102.56 (5.01)	0.100
*Klebsiella pneumoniae*	ATCC 33495	LO48	Loja	*Scaptotrigona problanca*	0.500 (0.061)	118.39 (14.53)	0.100
	KPC 609803	OR24.1	El Oro	*Melipona indecisa*	0.121 (0.017)	92.21 (12.94)	0.700

**Legend:** The table illustrates the average results of the optical density at 630 nm (A_630_) and biofilm biomass percentages (Biomass) with their standard deviation values (SD). All assays were realized in triplicate on different days. ^1^*P*-values of the treated biofilm with a certain honey sample when compared to the osmotic control through the Wilcoxon test.

### 3.3 Total cell count and cell viability on treated-biofilm samples

**Fig. 1:**
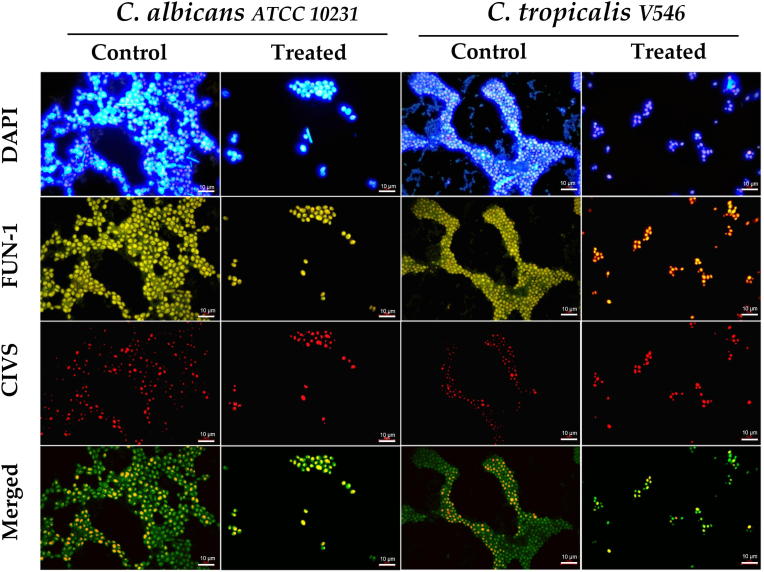
Illustration of the honey-treated and untreated 24-h biofilms of *C. albicans* and *C. tropicalis* by fluorescence microscopy (FM) analysis. DAPI staining was used for the total cell count, EPS bridges, and eDNA evaluation, while FUN-1 and CIVS dyes evaluated the cellular viability in yeast cells. When the plasma membrane was intact, yeast cells internalized FUN-1 showing diffuse green cytosolic fluorescence. However, only metabolically active yeast cells formed orange-red intravacuolar cylindrical structures (CIVS). The scale magnification is presented by a white line in the lower right corner indicating 10 μm. Imaging analysis used pictures with a total magnification of 1000x obtained by an Olympus BX50 microscope and then analyzed through AmScope software (version 1.2.2.10.). The merged images were processed by Fiji-ImageJ software (version 1.57). (For interpretation of the references to color in this figure legend, the reader is referred to the Web version of this article.)

**Fig. 2:**
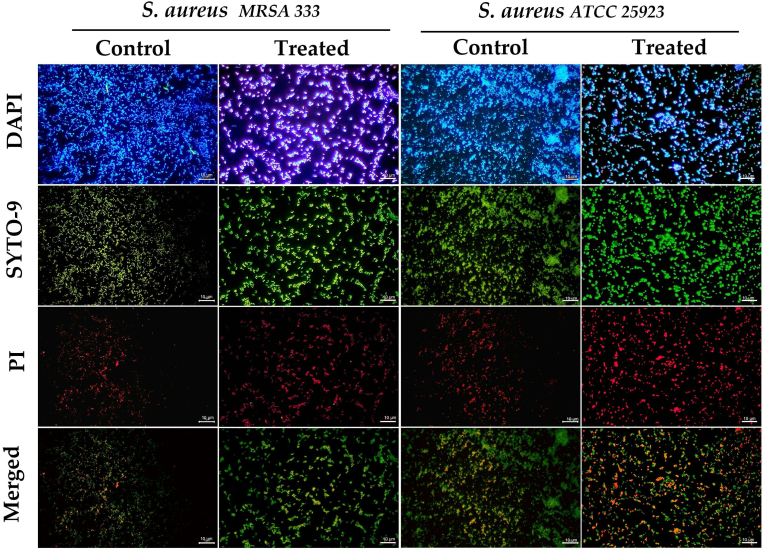
Illustration of the honey-treated and untreated 24-h biofilms of *S. aureus* MRSA 333 and ATCC 25923 by fluorescence microscopy (FM) analysis. DAPI staining was used for the total cell count, EPS bridges, and eDNA evaluation, while SYTO-9 and propidium iodide (PI) dyes evaluated the cellular viability in bacterial cells. SYTO 9 dye freely penetrated most cells with intact membranes, producing a green fluorescence, and PI dye only penetrated cells with damaged membranes, showing an intense red fluorescence. The scale magnification is presented by a white line in the lower right corner indicating 10 μm. Imaging analysis used pictures with a total magnification of 1000x obtained by an Olympus BX50 microscope and then analyzed through AmScope software (version 1.2.2.10.). The merged images were processed by Fiji-ImageJ software (version 1.57). (For interpretation of the references to color in this figure legend, the reader is referred to the Web version of this article.)

**Fig. 3:**
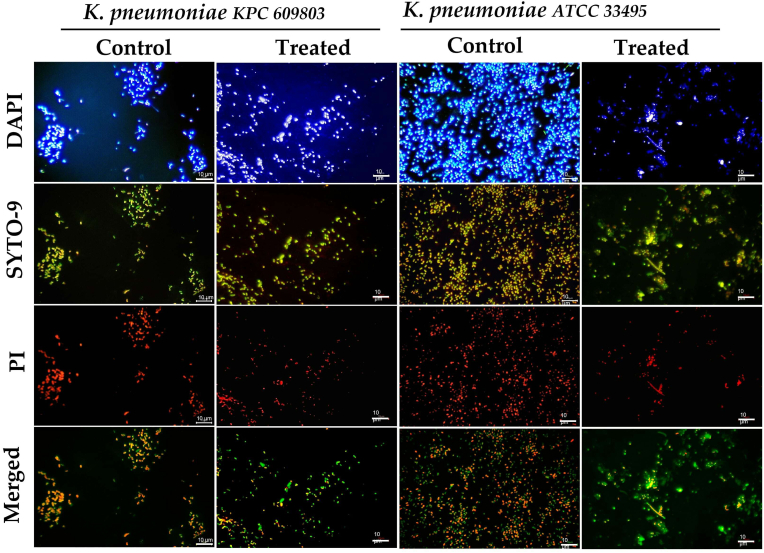
Illustration of the honey-treated and untreated 24-h biofilms of *K. pneumoniae* KPC 609803 and ATCC 33495 by fluorescence microscopy (FM) analysis. DAPI staining was used for the total cell count, EPS bridges, and eDNA evaluation, while SYTO-9 and propidium iodide (PI) dyes evaluated the cellular viability in bacterial cells. SYTO 9 dye freely penetrated most cells with intact membranes, producing a green fluorescence, and PI dye only penetrated cells with damaged membranes, showing an intense red fluorescence. The scale magnification is presented by a white line in the lower right corner indicating 10 μm. Imaging analysis used pictures with a total magnification of 1000x obtained by an Olympus BX50 microscope and then analyzed through AmScope software (version 1.2.2.10.). The merged images were processed by Fiji-ImageJ software (version 1.57). (For interpretation of the references to color in this figure legend, the reader is referred to the Web version of this article.)

**Fig. 4:**
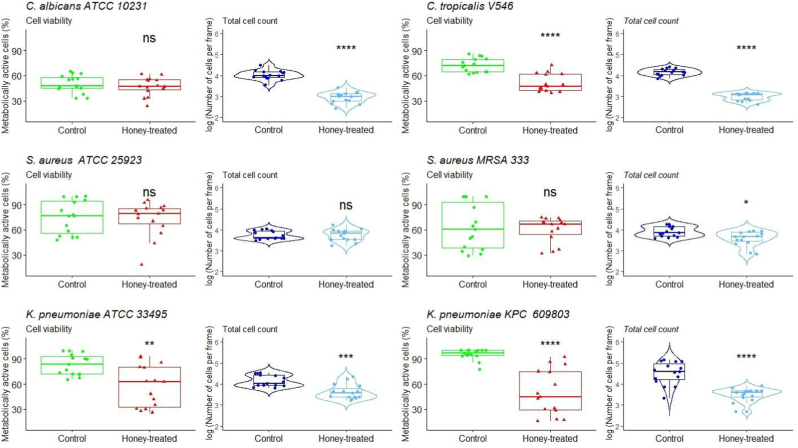
Graphical representation of the total cell count and cell viability results of honey-treated and untreated 24-h biofilms of the pathogens by fluorescence microscopy (FM) analysis. The total cell count and cell viability results of the microbial cells were calculated from the obtained images using Fiji ImageJ software (version 1.57). Non-parametric Wilcoxon tests were used to identify significant differences between control (untreated) and honey-treated samples, appearing in the plot as *P*-values >0.05 as nonsignificant P value (ns), * as *P*-values <0.05, ** as *P*-values <0.01, *** as *P*-values <0.001, and **** as *P*-values <0.0001. The present plot was realized in R studio software (version 4.0).

The FM analysis using DAPI (4',6-diamidino-2-phenylindole, dihydrochloride) and LIVE/DEAD staining allowed us to evaluate the honey set (OR24.1, LR34, LO40, LO48, and LO53) samples’ antibiofilm effect on the total cell count, cell viability, and extracellular polymeric substance (EPS) content in 24-h biofilms (see Supplementary Table S4). As aforementioned, a LIVE/DEAD Yeast Viability Kit was used for *Candida* species (see Fig. 1), while a LIVE/DEAD BacLight Bacterial Viability Kit was applied for *S. aureus* (see Fig. 2) and *K. pneumoniae* strains (see Fig. 3), allowing us to compare the inhibition biofilm assays between these different microorganisms. When looking at the compiled results in Fig. 4, the log reductions in the total cell count of honey-treated samples were statistically significant in all pathogens (*P*<0.05), apart from *S. aureus* ATCC 25923 (*P*=0.84), which did not show any reduction. When compared with untreated 24-h biofilms, the remaining microorganisms evidenced a log reduction of between 8.16 and 28.37%, being again *C. albicans* (26.55%) and *C. tropicalis* (28.37%) the most affected microorganisms, followed by *K. pneumoniae* KPC 609803 (22.39%), and *K. pneumoniae* ATCC 33495 (12.53%). Concerning the viability of the cells within the biofilm, only *C. tropicalis*, *K. pneumoniae* ATCC 33495, and *K. pneumoniae* KPC 609803 demonstrated significant drops in cell viability (*P* <0.01), decreasing by 21.67, 25.69, and 45.62%, respectively. Finally, a preliminary analysis of the content of EPS within the biofilm samples was carried out with ImageJ software through DAPI staining outside the cells in the collected pictures (grays units), showing a statistical EPS diminution in *C. albicans* (24.40%), *C. tropicalis* (34.09%), *K. pneumoniae* ATCC 33495 (34.72%) and *S. aureus* ATCC 25923 (51.76%) with *P*-values <0.01 (see Supplementary Materials).

### 3.4 Structural composition on treated-biofilm samples

**Fig. 5:**
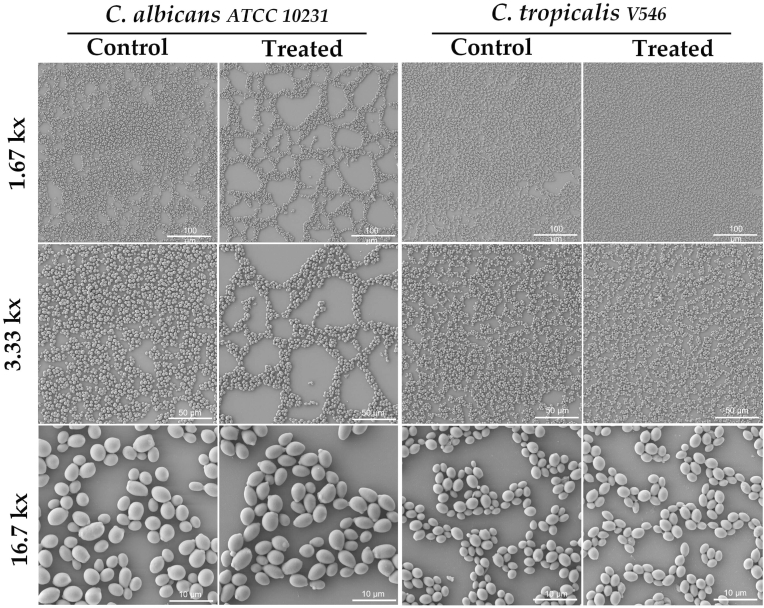
Illustration of the honey-treated and untreated 24-h biofilms of *Candida albicans* and *Candida tropicalis* by scanning electron microscopy (SEM) analysis at different magnifications (1.67, 3.33, and 16.7 kx). Tescan Mira 3 scanning electron microscope equipped with a Schottky field emission gun (Schottky FEG-SEM) was used for imaging evaluation. The area of yeast cells and other morphological parameters were calculated from the obtained images using Fiji ImageJ software (version 1.57). The scale magnification is shown by a white line in the lower right corner indicating 100 (at 1.67 kx), 50 (at 3.33 kx), and 10 μm (at 16.7 kx).

**Fig. 6:**
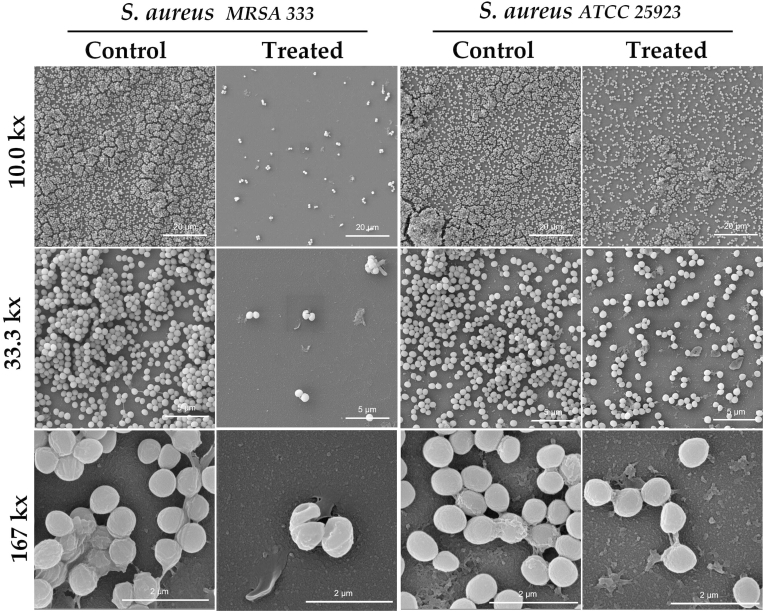
Illustration of the honey-treated and untreated 24-h biofilms of *S. aureus* MRSA 333 and ATCC 25923 by scanning electron microscopy (SEM) analysis at different magnifications (10.0, 33.3, and 167 kx). Tescan Mira 3 scanning electron microscope equipped with a Schottky field emission gun (Schottky FEG-SEM) was used for imaging evaluation. The area of bacterial cells and other morphological parameters were calculated from the obtained images using Fiji ImageJ software (version 1.57). The scale magnification is shown by a white line in the lower right corner indicating 20 (at 10.0 kx), 5 (at 33.3 kx), and 2 μm (at 167 kx).

**Fig. 7:**
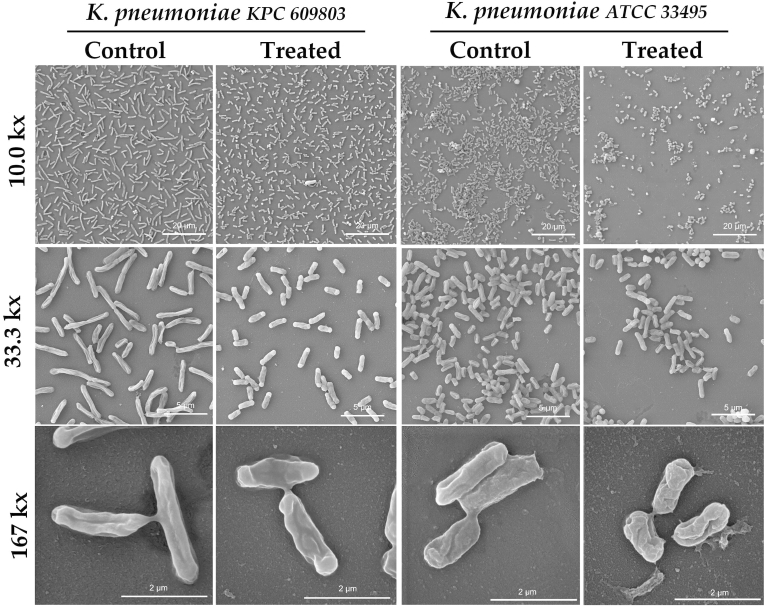
Illustration of the honey-treated and untreated 24-h biofilms of *K. pneumoniae* KPC 609803 and ATCC 33495 by scanning electron microscopy (SEM) analysis at different magnifications (10.0, 33.3, and 167 kx). Tescan Mira 3 scanning electron microscope equipped with a Schottky field emission gun (Schottky FEG-SEM) was used for imaging evaluation. The area of bacterial cells and other morphological parameters were calculated from the obtained images using Fiji ImageJ software (version 1.57). The scale magnification is shown by a white line in the lower right corner indicating 20 (at 10.0 kx), 5 (at 33.3 kx), and 2 μm (at 167 kx).

**Fig. 8:**
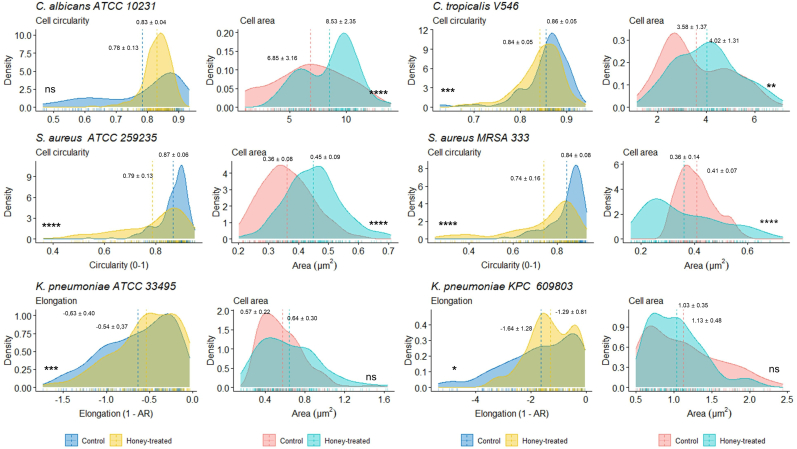
Graphical representation of the main cell morphological parameters of honey-treated and untreated 24-h biofilms of the pathogens by scanning electron microscopy (SEM) analysis. The area, circularity, and elongation parameters of the microbial cells were calculated from the obtained images using Fiji ImageJ software (version 1.57). Non-parametric Wilcoxon tests were used to identify significant differences between control (untreated) and honey-treated samples, appearing in the plot as *P*-values >0.05 as nonsignificant P value (ns), * as *P*-values <0.05, ** as *P*-values <0.01, *** as *P*-values <0.001, and **** as *P*-values <0.0001. The present plot was realized in R studio software (version 4.0).

The SEM analysis evaluated the antibiofilm effect of honey samples (OR24.1, LR34, LO40, LO48, and LO53) on the structural biofilm disruption through cell morphological parameters, such as the size, form, shape, and structure of the cells within the biofilm (see Supplementary Table S5). Three different magnifications were used for the imaging evaluation of the *Candida* species (1.67, 3.33, and 16.7 kx; see Fig. 5), *S. aureus* strains (10.0, 33.3, and 167 kx; see Fig. 6), and *K. pneumoniae* strains (10.0, 33.3, and 167 kx; see Fig. 7). This allowed us to study the general disposition of biofilm patterns by fractal dimension index (FDI) under different morphological parameters of the cells by area, circularity, and elongation. The FDI only showed statistical differences between treated and untreated biofilm samples in 3 of the 6 evaluated pathogens: *S. aureus* ATCC 25923 (*P* =0.019), *S. aureus* MRSA 333 (*P* =0.00099), and *K. pneumoniae* KPC 609803 (*P* =0.0038), suggesting a potential disruption of the biofilm pattern. The FDI pattern was reduced by 8.33% in treated *S. aureus* ATCC 25923, while *S. aureus* MRSA 333 and *K. pneumoniae* KPC 609803 showed an increment of 8.33 and 12.05% in the FDI pattern when compared to the untreated biofilm controls (see Supplementary Table S5), respectively. As shown in Fig. 8, when analyzing morphological parameters from cells within the biofilm, the cell area was statistically affected between treated and untreated biofilm samples among *Candida* species and *S. aureus* strains (*P* <0.01). *Candida albicans*, *S. aureus* ATCC 25923, and *C. tropicalis* showed the greatest increment of cell area values by 25.48, 18.42, and 9.29% when compared to the controls, respectively. However, *S. aureus* MRSA 333 suffered a reduction in the cell area of treated biofilm samples by 7.50%. It is also important to mention that in Fig. 8, cell circularity was selected on *Candida* species and *S. aureus* strains as a shape parameter, while elongation was prioritized on *K. pneumoniae* strains due to the morphological nature of the cells between these pathogens. Cell circularity was statistically affected between treated and untreated biofilm samples among *Candida tropicalis* and *S. aureus* strains (*P* <0.001) with the exception of *Candida albicans* (*P* =0.87), where cell circularity was reduced between 2.33 and 11.90%. Meanwhile, both *K. pneumoniae* ATCC 33495 and *K. pneumoniae* KPC 609803 showed statistical differences in their cell elongation between treated and untreated biofilm samples (*P* <0.05), demonstrating a similar reduction of 23.53 and 24.26% on treated biofilms, respectively. However, other size, form, and shape cell parameters were also found to be statistically significant among treated and untreated pathogens (see Supplementary Materials), evidencing the importance or usefulness of cell morphological analysis for biofilm samples.

## 4 Discussion

Ecuador has an enormous biodiversity in stingless bees evidencing a total of 132 species distributed in 23 genera found in 24 provinces accordingly to Vit and colleagues (Vit et al., 2018). In 2021, Villacrés-Granda and colleagues evaluated 26 multifloral honey samples of stingless bees from 5 Ecuadorian provinces from 3 geographical regions (Andean, Amazonian, and Coastal Pacific). Although Ecuador has more than 100 honey-producing stingless bee species, the most common stingless bees used by beekeepers in our previous study were *Melipona* and *Scaptotrigona* genera (Villacrés-Granda et al., 2021), in agreement with other study on stingless bee honey from the Ecuadorian Amazon (Guerrini et al., 2009). Therefore, the present study analyzed the potential antibiofilm activity of thirty-five different honey samples produced by ten diverse stingless bee species of five provinces of Ecuador (Tungurahua, Pastaza, El Oro, Los Ríos, and Loja) against susceptible and MDR strains of *Staphylococcus aureus* and *Klebsiella pneumoniae*, as well as *Candida albicans* and *Candida tropicalis*. The honey samples were evaluated against 24-h biofilms through biofilm biomass reduction, total cell count together with cell viability, and structural biofilm disruption. The ability to establish biofilm is a well-known property among several pathogens. It is a major virulence factor among primary or opportunistic infections due to several intrinsic factors, such as antimicrobial resistance, immune system evasion, and horizontal gene transfer (HGT) mechanisms in multispecies biofilms (Atiencia-Carrera et al., 2022a, 2022b; Cavalheiro and Teixeira, 2018; de Barros et al., 2020). Given the increase of antimicrobial resistance worldwide, alternative treatments have been extensively studied in the last decade. It is here where honey has been one of the natural products that has attracted the attention given its extensive and ancient use as a natural antimicrobial agent (Scepankova et al., 2017). Besides its potential as alternative treatment against MDR pathogens, honey can also prevent deteriorative oxidation reactions in foods (*e.g.*, the browning of fruit and vegetables and lipid oxidation in animal products) and inhibit the microbial growth of foodborne pathogens and food spoilage by them (Al-Waili et al., 2012). The main antimicrobial mechanisms of honey are related to the osmotic action caused by its high sugar content, as well as its acidity and hydrogen peroxide (H_2_O_2_) content (Proaño et al., 2021), and the action of other components present in honey such as polyphenols (Nolan et al., 2019). As indicated above, five honey samples showed the highest values of biofilm reduction in the inhibition tests against the six microorganisms, belonging to three specific provinces of Ecuador (Loja, El Oro, and Los Ríos; see Table 3). Considering the results of the analysis of some of the components that could influence the antibacterial activity of these honeys (pH, free and total acidity, total sugar content, minerals, amino acids, vitamin C, and certain organic acids) previously reported (Villacrés-Granda et al., 2021), no particularly distinctive factors were identified between honey samples. Villacrés-Granda et al. (2021) demonstrated the acidity and high-water content of Meliponine honey, where *Melipona* sp. evidenced more free amino acid content when compared to *Oxytrigona mellaria* which showed the highest content in proteins. Only 5 of the 12 stingless bee species' honey contained vitamin C, which was highest in *O. mellaria*. The most abundant minerals were the macronutrients, potassium, and calcium. These honey samples showed higher values of free acidity, whereby there was a significant correlation between free acidity and total organic acid content (*P* ≤ 0.01). Minimal inhibitory concentration (MIC) assays of honey samples inhibited *S. aureus* MRSA 333 and *K. pneumoniae* KPC 609803 (Villacrés-Granda et al., 2021). However, no particular organic acid or other bioactive compound was associated with their antibacterial activities. Thus, after the antibiofilm activity was validated in a more broad range of microbial pathogens, a characterization of the polyphenolic profile of these samples was carried out in the present study to identify other components that could justify the effect described here (Villacrés-Granda et al., 2021) and the previous study of Ng and colleagues that postulated phenolic and flavonoid compounds to be the only phytochemicals that contributed to the non-peroxide antibacterial activity of stingless bee honey (Ng et al., 2020). As indicated above, a wide variety of polyphenolic compounds were identified, which in many cases characterized the honeys of particular regions. An example of this was the presence of lignans (*e.g.,* medioresinol) only in the samples from the province of Loja. It has been reported that medioresinol possessed antibacterial effects against antibiotics-susceptible or antibiotics-resistant strains such as *Pseudomonas aeruginosa* (Hwang et al., 2013), suggesting its potential as a therapeutic agent and adjuvant for treatment of bacterial infection, an aspect to highlight in honeys that have this compound. In the present study, honey samples from Loja showed an effective antifungal activity in both biofilm inhibition and eradication assays against *C. albicans* and *C. tropicalis* strain evidencing properties that affect both planktonic and biofilm forms of these pathogens. Other compounds to highlight were the mere presence of compounds from the coumarin family only in the Los Rios samples. Recent studies demonstrated, for the first time, that scopoletin, one of the coumarins identified in the honey samples from Los Rios, acts as an effective antimicrobial phytocompound against *Staphylococcus aureus* MRSA 333 strain with properties that affect the initial formation their biofilms (Lemos et al., 2020). However, this antibacterial activity was not observed in mature biofilms of *Staphylococcus aureus* MRSA 333. Another group of predominant phytocompounds in these honeys were kaempferol and quercetin, either in glycosidic forms or as aglycones. Both flavonoids and its derivatives have been associated with important antimicrobial effects (Nguyen and Bhattacharya, 2022). In fact, kaempferol has been reported to inhibit the primary binding phase of biofilm formation in *Staphylococcus aureus*, while quercetin and its derivatives have been associated with important growth inhibitory factors of a large group of microorganisms such as: Methicillin-resistant (MRSA) and Methicillin-sensitive (MSSA) *Staphylococcus aureus*, *Pseudomonas aeruginosa*, *Micrococcus luteus*, *Shigella sonnei*, *Streptococcus mutans*, *Streptococcus sobrinus*, *Streptococcus sanguis*, *Lactobacillus acidophilus*, *Actinobacillus actinomycetemcomitan*s, and *Prevotella intermedia* (Nguyen and Bhattacharya, 2022). Notwithstanding the particularities indicated it was difficult to identify a pattern that would justify the marked antimicrobial effect of these five honey samples (LO40, LO48, LO53, OR24.1, and LR34) with respect to the commercial honey samples reported in several studies, which supports, in a certain way, the argument of the multifactorial nature of the antimicrobial activity of honey (Nolan et al., 2019; Proaño et al., 2021; Scepankova et al., 2017).

From the point of view of its antimicrobial activity, the effect of the honey samples (OR24.1 and LR34) against *S. aureus* strains (ATCC 25923 and MSRA 333) appeared in the superficial layers of the biofilm, as observed by SEM analysis (see Fig. 6), which was unable to reach more active and persistent cells located in deeper layers of the biofilm, as reported in previous studies (Lister and Horswill, 2014; E. Nishio et al., 2016; Reffuveille et al., 2017).

In the antibiofilm activity carried out in this study, no significant relationship was found between stingless bee species and any specific microorganism, aside from *C. albicans* and *C. tropicalis*, where honey samples from *Scaptotrigona problanca* demonstrated a significant antibiofilm activity*.* In general, little is known about the specificity of any honeybee species with a certain microorganism. However, some studies reported that honey produced by *Tetragonisca angustula* (Zamora et al., 2017) and *Trigona* spp. (Ng et al., 2017), and propolis produced by *Tetragonisca fiebrigi* and *Scaptotrigona jujuyensis* (Brodkiewicz et al., 2018) inhibited the biofilm formation of *S. aureus*, including MRSA strains, by 50–70% but were notoriously unable to eradicate preformed biofilms*.* In addition, combined treatments with *Tetragonisca angustula* honeys from Costa Rica and ampicillin or vancomycin allowed the antibiotics to regain their antimicrobial activities on a *Staphylococcus aureus* biofilm (Zamora et al., 2017). Concerning *K. pneumoniae* and *Candida* spp. biofilms, no literature is available for us to discuss, making them another novelty in this study to the authors' best knowledge. As previously indicated, little is known about stingless bee honey’ antibiofilm activity and the few recent studies reported different bioactive compounds from a diverse set of honey-producing bee species with a different floral origin (Ávila et al., 2019a; Rosli et al., 2020; Sousa et al., 2016), and even with a distinct honey-associated microbiome (Ávila et al., 2019b; Baharudin et al., 2021; Julika et al., 2019; Mohammad et al., 2020; Ngalimat et al., 2019; Zulkhairi Amin et al., 2019).

By FM analysis, we were able to confirm a reduction of EPS produced by all biofilms (see Supplementary Table S4). Concerning *C. albicans* and *C. tropicalis*, it can be assumed that the efficacy of our honey samples (LO40 and LO53) on these opportunistic species, when compared with non-treated samples (see Fig. 1)*,* is probably associated with a multifactorial and synergic combination of flavonoids, phenolic acids, and perhaps antimicrobial peptides (Alvarez-Suarez et al., 2018). Contrasting to bacterial pathogens, these *Candida* species demonstrated statistically specific bee-associated species features with antimicrobial properties. Therefore, potential specific antimicrobial peptides together with a certain chemical composition of these phenolic acids, flavonoids, and triterpenes could synergically alter the normal metabolism of this fungus and consequently cell viability (Candiracci et al., 2011; Canonico et al., 2014; Estevinho et al., 2011; Liberio et al., 2011; Maghfiroh et al., 2021). However, the lack of quantitative chemical and peptide composition represents a limitation of the present work that should be clarified in future studies. It is important to mention that cell viability did not show statistical differences in *C. albicans* biofilms. Further studies should also be improved the evaluation of viability cell data through the fluorescent probe-based methodology (i.e., the LIVE/DEAD Yeast Viability Kit), as widely discussed by previous studies (Atiencia-Carrera et al., 2022b; Netuschil et al., 2014; Welch et al., 2012).

Last, but not least, we performed SEM analysis and further evaluated the structural biofilm disruption and cell morphological parameters, evidencing statistically significant differences among treated and non-treated pathogens, in agreement with recent studies involving other biofilm-forming microorganisms, such as *S. pyogenes*, *P. aeruginosa, Streptococcus pneumoniae* (Alkafaween et al., 2021a, 2021b), and *C. albicans* (Hau-Yama et al., 2020). However, it is worth noting that the present study performed a more exhaustive analysis of cell morphologies when compared with the previous studies. Besides the morphological alterations reported in this study, some studies describe the presence of protoplasts, spheroplasts, and septa in treated *S. aureus* strains due to cell wall weakening by peptidoglycan degradation and inhibition of bacterial cell division (Cushnie et al., 2016; Domingos et al., 2021; Nishio et al., 2016). These inhibitory mechanisms on *S. aureus* biofilms previously reported could explain the differences observed in cell circularity on treated biofilms of the present study as “deflated balloon-like forms folded on itself” in MRSA strains, probably due to osmotic lysis enhanced by honey flavonoids (Cushnie and Lamb, 2005; Ouyang et al., 2018; Proaño et al., 2021). On the other hand, Jenkins et al. (2011) proposed that the presence of septa and large elongated cells was a consequence of the flavonoid-mediated inhibition of murein hydrolase, which is necessary for bacterial cleavage, as observed in both *S. aureus* strains (Jenkins et al., 2011; Ouyang et al., 2018). In 2017, Ng et al. (2017) reported longer rod and filamentous forms, suggesting inhibition of cell septation and cell division when treating *E. coli* with *Heterotrigona itama* honey. However, in the present study, both treated *K. pneumoniae* strains showed shortened rod and filamentous forms. Moreover, regarding the antifungal effect on *C. albicans*, the identified changes were regarding the size regularity and morphology of the membrane and similar findings were also observed with *Melipona becchei* honey (Hau-Yama et al., 2020) and Jujube honey (Ansari et al., 2013). Although several studies have confirmed that honey's antifungal effect is strongly linked to the floral and entomological origin (Alvarez-Suarez et al., 2018; Boorn et al., 2010; Fernandes et al., 2021; Irish et al., 2006; Morroni et al., 2018; Ramón-Sierra et al., 2019; Suntiparapop et al., 2015; Zamora et al., 2015), little is known about the antifungal properties of stingless bee honeys. Several studies have found that some phytochemical compounds, especially terpenes and flavonoids, present in natural products including honey, can inhibit morphological transitions in *Candida* species (Al-Ghanayem, 2022; Ansari et al., 2013; Calixto Júnior et al., 2015; da Silva et al., 2021; Prasath et al., 2020; Priya and Pandian, 2022; Soliman et al., 2017). In our case, the evidence obtained was not sufficient to reinforce this idea. In addition, we found a statistically significant increase in yeast area in both *Candida* species treated with honey, probably due to the availability of sugars. Furthermore, FM analysis evidenced a reduction in viability and cell count, which could explain a possible antagonistic effect between the antifungal effect exerted by osmotic pressure and the amount of useable sugars, because these pathogens adapt their metabolism according to the available nutrients by different sugar-sensing systems (Ng et al., 2016; Pemmaraju et al., 2016; Van Ende et al., 2019; Weerasekera et al., 2018). The osmotic control used in the present study is formed by sucrose, maltose, fructose and glucose as predominant sugars on honey samples accordingly to Cooper and colleagues (Cooper et al., 2002). Therefore, we assumed that these predominant honey sugars are responsible to activate *Candida* sugar-sensing systems and could be responsible for the increase in yeast area in the present study. However, other minority sugars should also contribute to *Candida* species' resilience against the antimicrobial activity of stingless bee honey. Fermentable sugars such as sucrose, fructose, glucose and maltose promoted adhesion, colonization, and biofilm development of *Candida* species, such as *C. albicans* and *C. tropicalis*, on both biotic and abiotic surfaces (Weerasekera et al., 2018). In *C. albicans*, the glucose transporter gene family was first studied, and it can be divided into *CaHXT* and *CaSNF3* groups. The sensing of fructose and mannose induces the expression of 7 of the 20 hexose transporters genes including *CaHGT12* and *CaHGT*7 (Van Ende et al., 2019). However, several sugar-sensing systems remains unknown or uncharacterized in *C. albicans* and mostly non-albicans *Candida* (NAC) species, such as *C. tropicalis*. Rodaki and colleagues demonstrated that Cap1 and Hog1 probably mediate glucose-enhanced resistance to oxidative stress in *C. albicans* (Rodaki et al., 2009). Meanwhile, Suchodolski and Krasowska evidenced that fructose can simultaneously increase the activation of Cdr1p (membrane efflux transporter) and Mdr1p (major facilitator superfamily transporter) in *C. albicans* (Suchodolski and Krasowska, 2021). Recently, Ke and colleagues evidenced that *C. tropicalis* wild-type strain exhibited normal growth to different carbon sources, including glucose, sucrose, galactose, and glycerol (Ke et al., 2023). Like *C. albicans*, the AMP-activated protein kinase (AMPK) pathway in *C. tropicalis* is responsible for the metabolism of nonglucose carbon sources and is involved in cell wall integrity and virulence (Ke et al., 2023). So, further studies should characterize the phenotypic shift of *Candida*-related biofilms when treated with stingless bee honey samples.

A new morpho-structural parameter was exploratorily evaluated in the present study, namely the fractal dimension index (FDI). FDI can be considered a structural indicator of the complexity and stability of the biofilm, as well as the degree of response to changes in the environment, allowing us to evaluate biomass variations and changes in surface roughness, cell distribution pattern, and the level of fragmentation (Artyushkova et al., 2015; Grzegorczyk et al., 2018; Kassinger and van Hoek, 2020; Qin et al., 2021). Even so, no statistical differences were found between control and treated samples in both *Candida* species and *K. pneumoniae* ATCC 33495. Both *S. aureus* strains and *K. pneumoniae* KPC 609803 evidenced statistical differences in FDI between treated and non-treated biofilms (see Supplementary Table S5). Nonetheless, to better understand this type of structural biofilm evaluation, further studies are necessary to realize 3D biofilm architecture analysis through atomic force microscopy (AFM) and/or confocal laser scanning microscopy (CLSM), as in other studies (Grzegorczyk et al., 2018).

In summary, the present study provided more detailed information on the antibiofilm activity of stingless bee honey samples against *S. aureus*, *K. pneumoniae*, and *Candida* species, evidencing the ability of biofilm inhibition through biomass, total cell count and viability, and cell morphological parameters. However, this study has several limitations. For example, the antibiofilm activity was only studied in monospecies biofilms and the absence of analyses based on metabolic or gene expression, flow cytometry, confocal microscopy, and quantitative polymerase chain reaction to assess the differences between control and treated biofilms. Future studies should implement these new analyses in the present biofilm evaluation and develop polymicrobial biofilm models to provide a more detailed picture of the antibiofilm effects of stingless bee honeys (Kucera et al., 2014; Sun et al., 2008; Woods et al., 2012).

## 5 Conclusions

This study achieved its objective to demonstrate the antibiofilm activity of stingless bee honeys against gram-positive, gram-negative, and yeast pathogens, showing biofilm inhibition of 63–80% in biomass, a significant reduction in the total cell account and viability, as well as differences in cell morphological parameters by SEM analysis. On the other hand, the approach of the multifactorial character of the antimicrobial activity of honey is also reinforced. To the authors' best knowledge, this is the first study to simultaneously analyze biofilms of susceptible and multidrug-resistant strains of *S. aureus* and *K. pneumoniae*, as well as different *Candida* species by biomass assays, fluorescence microscopy, and scanning electron microscopy. We were able to validate antibiofilm activity by several stingless bee honey types from different provinces of Ecuador. The main shortcomings of the present study include the lack of information on the floral origin of each honey sample per stingless bee species, quantification of each polyphenol content, and phenotypic shift expression of the pathogens-treated biofilms. Further studies should analyze the molecular and metabolic network that influences the inhibition of the biofilm formed by different pathogens by stingless bee honeys and correlate the stingless bee honeys’ floral origin and polyphenol content with their antimicrobial activities. Stingless bee honeys from Ecuador are a promising candidate for the research and development of novel antibiofilm molecules for the treatment of multidrug-resistant bacterial infections and clinically important fungal infections.

## Funding

This work was supported by Universidad San Francisco de Quito (Ecuador) through El Politécnico grant under the Project ID: 17578 entitled “*Estudio de los parámetros de calidad, composición química y actividad biológica de la miel, polen y pan de abeja de diferentes orígenes de la región andina de Ecuador*” and COCIBA research budget under the Project ID: 17357 entitled “*Alternative approaches for eliminating Biofilms*”. The funders had no role in study design, data collection, analysis, decision to publish, or composition of the manuscript. The APC was funded by the Research Office of Universidad San Francisco de Quito (USFQ).

## CRediT authorship contribution statement

**Fausto Sebastián Cabezas-Mera:** Software, Formal analysis, Investigation, Data curation, Writing – original draft, preparation, Writing – review & editing, Visualization. **María Belén Atiencia-Carrera:** Software, Formal analysis, Investigation, Data curation, Writing – original draft, preparation, Writing – review & editing, Visualization. **Irina Villacrés-Granda:** Conceptualization, Methodology, Writing – review & editing, Visualization. **Adrian Alexander Proaño:** Methodology, Formal analysis, Writing – review & editing, Visualization. **Alexis Debut:** Methodology, Validation, Formal analysis, Resources, Writing – review & editing, Visualization. **Karla Vizuete:** Validation, Formal analysis. **Lorena Herrero-Bayo:** Formal analysis, Investigation, Writing – review & editing. **Ana M. Gonzalez-Paramás:** Methodology, Validation, Writing – review & editing. **Francesca Giampieri:** Formal analysis, Data curation, Writing – review & editing. **Reinier Abreu-Naranjo:** Investigation, Writing – review & editing. **Eduardo Tejera:** Conceptualization, Software, Writing – review & editing, Visualization, Project administration. **José M. Álvarez-Suarez:** Conceptualization, Methodology, Validation, Writing – review & editing, Visualization, Supervision, Project administration, Funding acquisition. **António Machado:** Conceptualization, Methodology, Validation, Resources, Writing – original draft, preparation, Writing – review & editing, Visualization, Supervision, Project administration, Funding acquisition.

## Declaration of competing interest

The authors declare that they have no known competing financial interests or personal relationships that could have appeared to influence the work reported in this paper.

## Data Availability

Data will be made available on request.
