# Supplementary material for: Evaluation of the polyphenolic profile of native Ecuadorian stingless bee honeys (Tribe: Meliponini) and their antibiofilm activity on susceptible and multidrug-resistant pathogens: An exploratory analysis
Source: Curr Res Food Sci. 2023 Jun 29;7:100543. doi: 10.1016/j.crfs.2023.100543 (PMC10344713; doi:10.1016/j.crfs.2023.100543)
Supplement: Multimedia component 1 [file mmc1.docx]

# **Supplementary Materials**

**Supplementary File S1.** Database of the biomass reduction analysis of honey-treated biofilms through optical density assays at 630 nm (A_630_).


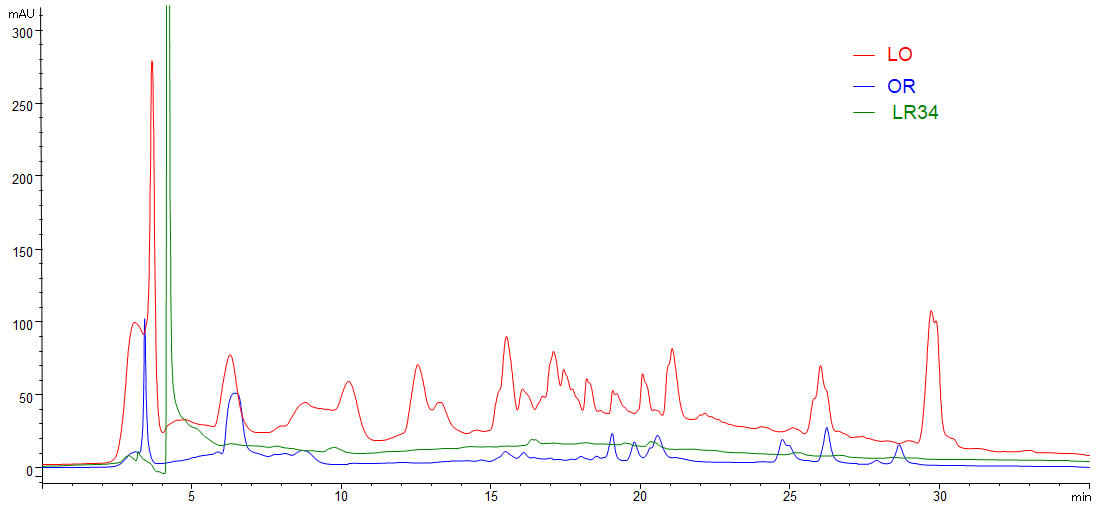


**Supplementary Figure S1**. Representative chromatograms, recorded at 360 nm, corresponding to methanolic extracts from honeys LO, OR and LR.

**Supplementary Table S1.** Summary of the information of honey sample set of the present study previously collected by Villacrés-Granda et al. (2021).

| **#** | **Sample Code** | **Stingless bee species** | **Region** | **Province** | **Region or place** | **Amount of sample (g)** | **Collection date** | **Season & temperature (ºC)** | **Seasonal precipitation (mm)** |
| --- | --- | --- | --- | --- | --- | --- | --- | --- | --- |
| 1 | RN9 | Unidentified | Andean | Tungurahua | Río Negro | 17 | 19/11/2019 | Rainy season (12.09ºC) | 379.13 |
| 2 | OR26 | *Melipona indecisa* | Coastal | El Oro | Saracay | 19 | 06/09/2018 | Dry season (21.36ºC) | 379.13 |
| 3 | OR28.1 | *Scaptotrigona problanca* | Coastal | El Oro | Saracay | 21 | 06/09/2018 | Dry season (21.36ºC) | 379.13 |
| 4 | OR15M | *Cephalotrigona* sp. | Coastal | El Oro | Saracay | 21 | 15/12/2018 | Rainy season (21.74ºC) | 392.57 |
| 5 | OR27.2 | *Scaptotrigona problanca* | Coastal | El Oro | Saracay | 21 | 06/09/2018 | Dry season (21.36ºC) | 379.13 |
| 6 | PS10 | *Tetragonisca angustula* | Amazonian | Pastaza | Puyo | 21 | 24/08/2018 | Dry season (24.59ºC) | 1074.14 |
| 7 | OR26.1 | *Scaptotrigona problanca* | Coastal | El Oro | Saracay | 22 | 06/09/2018 | Dry season (21.36ºC) | 379.13 |
| 8 | RN7 | Unidentified | Andean | Tungurahua | Río Negro | 22 | 31/12/2018 | Rainy season (12.28ºC) | 392.57 |
| 9 | OR59M | *Scaptotrigona problanca* | Coastal | El Oro | Huacas-Balsas | 23 | 08/12/2018 | Rainy season (21.74ºC) | 392.57 |
| 10 | OA02 | Unidentified | Amazonian | Pastaza | Santa Rosa | 26 | 01/12/2019 | Dry season (25.70ºC) | 467.34 |
| 11 | PS08 | *Melipona grandis* | Amazonian | Pastaza | Puyo | 29 | 24/08/2018 | Dry season (24.59ºC) | 1074.14 |
| 12 | OR16.1 | *Melipona mimetica* | Coastal | El Oro | Saracay | 30 | 15/12/2018 | Rainy season (21.74ºC) | 392.57 |
| 13 | OR24.1 | *Melipona indecisa* | Coastal | El Oro | Saracay | 30 | 15/12/2018 | Rainy season (21.74ºC) | 392.57 |
| 14 | OR22.1 | *Melipona indecisa* | Coastal | El Oro | Saracay | 30 | 06/09/2018 | Dry season (21.36ºC) | 379.13 |
| 15 | OR25.1 | *Melipona indecisa* | Coastal | El Oro | Saracay | 32 | 15/12/2018 | Rainy season (21.74ºC) | 392.57 |
| 16 | OR23M | *Melipona indecisa* | Coastal | El Oro | Saracay | 33 | 15/12/2018 | Rainy season (21.74ºC) | 392.57 |
| 17 | OA04 | Unidentified | Amazonian | Pastaza | Santa Rosa | 34 | 01/12/2019 | Dry season (25.70ºC) | 467.34 |
| 18 | LR41 | *Melipona cramptoni* | Coastal | Los Ríos | Quevedo | 37 | 01/06/2019 | Dry season (20.82ºC) | 463.10 |
| 19 | LR39 | *Melipona cramptoni* | Coastal | Los Ríos | Quevedo | 44 | 01/06/2019 | Dry season (20.82ºC) | 463.10 |
| 20 | LR33 | *Melipona cramptoni* | Coastal | Los Ríos | Quevedo | 48 | 01/06/2019 | Dry season (20.82ºC) | 463.10 |
| 21 | PS20 | *Melipona grandis* | Amazonian | Pastaza | Santa Clara | 48 | 28/08/2018 | Dry season (24.59ºC) | 1074.14 |
| 22 | LR35 | *Melipona cramptoni* | Coastal | Los Ríos | Quevedo | 50 | 01/06/2019 | Dry season (20.82ºC) | 463.10 |
| 23 | LR40 | *Melipona cramptoni* | Coastal | Los Ríos | Quevedo | 51 | 01/06/2019 | Dry season (20.82ºC) | 463.10 |
| 24 | PS18 | *Tetragonisca angustula* | Amazonian | Pastaza | Tarqui | 54 | 18/08/2018 | Dry season (24.59ºC) | 1074.14 |
| 25 | LR36 | *Melipona cramptoni* | Coastal | Los Ríos | Quevedo | 55 | 01/06/2019 | Dry season (20.82ºC) | 463.10 |
| 26 | LO40 | *Scaptotrigona problanca* | Andean | Loja | Balsa Real | 62 | 28 until 30/11/2018 | Rainy season (21.36ºC) | 379.13 |
| 27 | PS19 | *Tetragonisca angustula* | Amazonian | Pastaza | Tarqui | 63 | 18/08/2018 | Dry season (24.59ºC) | 1074.14 |
| 28 | LR34 | *Melipona* sp. | Coastal | Los Ríos | Quevedo | 87 | 01/06/2019 | Dry season (20.82ºC) | 463.10 |
| 29 | LO52 | *Scaptotrigona problanca* | Andean | Loja | El Guabo | 110 | 28 until 30/11/2018 | Rainy season (21.36ºC) | 379.13 |
| 30 | LR37 | *Melipona* sp. | Coastal | Los Ríos | Quevedo | 117 | 01/06/2019 | Dry season (20.82ºC) | 463.10 |
| 31 | LO53 | *Scaptotrigona problanca* | Andean | Loja | El Guabo | 137 | 28 until 30/11/2018 | Rainy season (21.36ºC) | 379.13 |
| 32 | PS16 | *Melipona nigrifacies* | Amazonian | Pastaza | Puyo | 158 | 19/08/2018 | Dry season (24.59ºC) | 1074.14 |
| 33 | LO48 | *Scaptotrigona problanca* | Andean | Loja | El Guabo | 175 | 28 until 30/11/2018 | Rainy season (21.36ºC) | 379.13 |
| 34 | PS15 | *Melipona fuscopilosa* | Amazonian | Pastaza | Puyo | 181 | 19/08/2018 | Dry season (24.59ºC) | 1074.14 |
| 35 | LO43 | *Scaptotrigona problanca* | Andean | Loja | Balsa Real | 231 | 28 until 30/11/2018 | Rainy season (21.36ºC) | 379.13 |

Legend- The average seasonal mean temperature and seasonal precipitation of the honey collection was based on the information obtained from World Bank Group on worldbank.org database ([https://climateknowledgeportal.worldbank.org/country/ecuador/climate-data-historical#](https://climateknowledgeportal.worldbank.org/country/ecuador/climate-data-historical), accessed on May 24^th^ 2023).

**Supplementary Table S2.** Mass spectral data and tentative identification of the compounds detected in the analysed samples.

|  | **MS**  **[M - H]^-^**  **(m/z)** | **MS^2^**  **[M - H]^-^**  **(m/z)** | **Tentative identity** | | |  |
| --- | --- | --- | --- | --- | --- | --- |
| **Phenolic family** |  |  |  | | |  |
| Lignans | 387 | 207, 163, 119 | Medioresinol | | |  |
| Coumarins | 677 | 485, 353, 191 | Scopoletin-hexose-pentose derivative | | |  |
|  | 314 | 191 | Scopoletin | | |  |
|  | 497 | 335 | Coumarin (e.g. dicoumarin) glycoside | | |  |
|  | 339 | 248, 191, 147 | Aesculin | | |  |
|  | 347 | 185 | Psoralen derivative | | |  |
| Phenolic acids | 179 | 135 | Caffeic acid | | |  |
|  | 181 | 163, 135, 119 | Dihydrocaffeic acid | | |  |
|  | 503 | 341, 179 | Caffeoyl-dihexose | | |  |
|  | 295 | 179, 133 | Caffeic acid cinnamyl ester | | |  |
|  | 355 | 193, 163 | Feruloylhexose | | |  |
|  | 529 | 511, 353, 179, 143 | Feruloylcaffeoylquinic acid | | |  |
|  | 327 | 165, 147 | Dihydrocoumaroylhexose | | |  |
|  | 223 | - | Sinapic acid | | |  |
|  | 137 | - | Hydroxybenzoic acid | | |  |
|  | 153 | 137, 121 | Protocatechuic acid | | |  |
|  | - | 197 | Syringic acid derivative | | |  |
|  | 167 | - | Dihydroxyphenylacetic acid | | |  |
|  | 151 | 137 | Hydroxyphenylacetic acid | | |  |
|  | 165 | - | Phenyl-lactic acid | | |  |
|  | 263 | 219, 153 | Abscisic acid | | |  |
|  | 331 | 287, 244 | Carnosic acid | | |  |
|  | 631 | 359 | Rosmarinic acid derivative | | |  |
|  | 615 | 359 | Rosmarinic acid derivative | | |  |
| **Flavonoids** | | | | |  |  |
| Flavones | 285 | 241, 175 | Luteolin | | |  |
|  | 299 | - | Chrysoeriol | | |  |
|  | 343 | 235, 285, 191 | Methoxyflavone (e.g. cirsilineol, eupatorin) | | |  |
| Flavanones | 463 | 301 | Hesperetin hexoside | | |  |
|  | 447 | 285 | Sakuranetin glycoside | | |  |
|  | 285 | 257, 195, 185 | Sakuranetin | | |  |
|  | 287 | - | Eriodictyol | | |  |
|  | 271 | - | Pinobanksin | | |  |
| Flavonols | 607 | 431, 285 | Kaempferol-deoxyhexose-glucuronide | | |  |
|  | 755 | 609, 447, 285 | Kaempferol-*O*-sophorose-*O*-rhamnoside | | |  |
|  | 755 | 593, 285 | Kaempferol-*O*-hexose-*O*-rutinoside | | |  |
|  | 739 | 575, 285 | Kaempferol-O-neohesperidose-rhamnoside | | |  |
|  | 593 | 429, 285 | Kaempferol-*O*-neohesperidoside | | |  |
|  | 593 | 285 | Kaempferol-*O*- rutinoside | | |  |
|  | 579 | 447, 285 | Kaempferol-*O*-hexose-*O*-pentoside | | |  |
|  | 447 | 285 | Kaempferol-*O*-hexoside | | |  |
|  |  |  | Kaempferol-*O*-rhamnoside | | |  |
|  | 739 | 577, 431, 415, 299 | | Methylkaempferol-*O*-rutinose-*O*-pentoside | | |
|  | 431 | 415, 299 | Methylkaempferol-pentoside | | |  |
|  | 285 | 257, 229, 195, 185 | Kaempferol | | |  |
|  |  |  |  | | |  |
|  |  |  |  | | |  |
|  | 609 | 301 | Quercetin-*O*-rutinoside | | |  |
|  | 447 | 301 | Quercetin-O-rhamnoside | | |  |
|  | 463 | 301 | Quercetin-O-hexoside | | |  |
|  | 345 | 301 | Hydroxyethylquercetin | | |  |
|  | 301 | - | Quercetin | | |  |
|  |  |  |  | | |  |
|  | 785 | 623, 315 | Methylquercetin (i.e. Isorhamnetin)-*O*- rutinoside-*O*-hexoside | | |  |
|  | 623 | 461, 315 | Isorhamnetin-*O*-rutinoside | | |  |
|  | 639 | 459, 477, 315 | Isorhamnetin-*O*-hexosyl(1→2)-hexoside | | |  |
|  | 477 | 315 | Isorhamnetin-*O*-hexoside | | |  |
|  |  |  | Isorhamnetin-*O*-glucuronide | | |  |
|  | 315 | 301 | Isorhamnetin | | |  |
|  | 315 | 301 | Tamarixetin | | |  |
|  | 491 | 329 | Dimethylquercetin-*O*-hexoside | | |  |
|  | 329 | - | Dimethylquercetin | | |  |
| Chalcone | 435 | 315, 273, 145 | Phlorizin | | |  |
| Isoflavones | 281 | - | Pseudobaptigenin | | |  |
| Others | 241 | 198 | Lumicrome | | |  |

**Supplementary Table S3**. Content of Total Phenolics (Folin-Ciocalteu method) expressed as gallic acid equivalents (GAE).

| **Honey Sample** | **mg GAE / 100 g of honey (SD)** |
| --- | --- |
| OR24.1 | 54.53 (0.77) |
| LR34 | 36.60 (1.38) |
| LO40 | 171.96 (3.16) |
| LO48 | 144.50 (3.64) |
| LO53 | 109.44 (3.10) |

**Supplementary Table S4.** Evaluation of the antibiofilm effect of honey samples on the total cell count, cell viability, and extracellular polymeric substances (EPS) content in 24-hour biofilms through fluorescence microscopy (FM) evaluation.

| Microorganism | Parameters | Control,  Mean (SD) | Honey-treated,  Mean (SD) | Pairwise comparison ^1^,  *P*-values |
| --- | --- | --- | --- | --- |
| *C. albicans* ATCC 10231 | Total cell count, log (cells/frame) | 4.03 (0.23) | 2.96 (0.26) | **3.4e-06** |
|  | Surface cell count, log (cells/cm^2^) | 7.35 (0.23) | 6.28 (0.26) | **3.4e-06** |
|  | Viability, % (live cells) | 50.12 (10.62) | 47.09 (10.79) | 0.43629 |
|  | Live/dead ratio, log (ratio) | 0.002 (0.190) | -0.055 (0.198) | 0.44242 |
|  | EPS content (grays units) | 77.95 (43.43) | 58.93 (33.53) | **0.00202** |
| *C. tropicalis* V546 | Total cell count, log (cells/frame) | 4.16 (0.16) | 2.98 (0.18) | **3.4e-06** |
|  | Surface cell count, log (cells/cm^2^) | 7.48 (0.16) | 6.30 (0.18) | **3.3e-06** |
|  | Viability, % (live cells) | 72.55 (8.29) | 50.88 (10.79) | **6.5e-06** |
|  | Live/dead ratio, log (ratio) | 0.440 (0.192) | 0.018 (0.194) | **5.2e-05** |
|  | EPS content (grays units) | 66.71 (27.86) | 43.97 (14.81) | **1.7e-11** |
| *S. aureus*  ATCC 25923 | Total cell count, log (cells/frame) | 3.75 (0.20) | 3.76 (0.28) | 0.84 |
|  | Surface cell count, log (cells/cm^2^) | 7.07 (0.20) | 7.08 (0.28) | 0.81941 |
|  | Viability, % (live cells) | 75.32 (19.59) | 73.19 (20.47) | 0.87 |
|  | Live/dead ratio, log (ratio) | 0.845 (0.943) | 0.517 (0.481) | 0.87 |
|  | EPS content (grays units) | 54.70 (9.49) | 26.39 (8.97) | **<2e-16** |
| *S. aureus*  MRSA 333 | Total cell count, log (cells/frame) | 3.92 (0.24) | 3.60 (0.36) | **0.028** |
|  | Surface cell count, log (cells/cm^2^) | 7.24 (0.24) | 6.92 (0.36) | **0.02789** |
|  | Viability, % (live cells) | 63.46 (27.32) | 60.45 (15.04) | 0.967 |
|  | Live/dead ratio, log (ratio) | 0.614 (1.067) | 0.194 (0.277) | 0.967 |
|  | EPS content (grays units) | 72.78 (13.45) | 91.15 (19.51) | **6.1e-13** |
| *K. pneumoniae*  ATCC 33495 | Total cell count, log (cells/frame) | 4.15 (0.27) | 3.63 (0.35) | **0.00053** |
|  | Surface cell count, log (cells/cm^2^) | 7.47 (0.27) | 6.94 (0.35) | **0.00053** |
|  | Viability, % (live cells) | 82.99 (12.38) | 57.30 (24.56) | **0.00367** |
|  | Live/dead ratio, log (ratio) | 0.960 (0.755) | 0.186 (0.533) | **0.00479** |
|  | EPS content (grays units) | 34.79 (17.31) | 22.71 (11.81) | **7.4e-13** |
| *K. pneumoniae*  KPC 609803 | Total cell count, log (cells/frame) | 4.51 (0.53) | 3.50 (0.31) | **4.8e-05** |
|  | Surface cell count, log (cells/cm^2^) | 7.83 (0.53) | 6.82 (0.31) | **4.8e-05** |
|  | Viability, % (live cells) | 95.31 (6.36) | 49.69 (26.85) | **9.0e-06** |
|  | Live/dead ratio, log (ratio) | 2.186 (1.308) | 0.013 (0.573) | **9.1e-06** |
|  | EPS content (grays units) | 29.60 (12.98) | 37.69 (17.31) | **0.00088** |

**Legend:** ^1^ Pairwise comparisons were realized through the Wilcoxon test. Evaluation of the *in vitro* antimicrobial effect of honey samples on fluorescence-based parameters in 24-hour biofilms. Non-parametric Wilcoxon tests were used to identify significant differences (*P*-values <0.05). The results of the analysis presented in this table were performed in Fiji-ImageJ (version 1.57).

**Supplementary Table S5.** Evaluation of the antibiofilm effect of honey samples on the cell morphological parameters in 24-hour biofilms through scanning electron microscopy (SEM) analysis.

| Microorganism | Cell morphological parameters | | Control,  Mean (SD) | Honey-treated,  Mean (SD) | Pairwise comparison ^3^,  *P*-values |
| --- | --- | --- | --- | --- | --- |
| *C. albicans* ATCC 10231 | Size | Length, µm | 2.95 (0.80) | 3.42 (0.71) | **1.90E-05** |
|  |  | Cell area, µm^2^ | 6.83 (3.20) | 8.57 (2.35) | **1.40E-06** |
|  | Form | Aspect ratio (AR), width/length | 1.33 (0.20) | 1.41 (0.16) | **2.90E-08** |
|  |  | Elongation, 1- AR | -0.32 (0.20) | -0.42 (0.15) | **2.90E-08** |
|  | Shape | Perimeter, µm | 10.23 (3.05) | 11.30 (1.70) | **0.00019** |
|  |  | Circularity^1^ | 0.78 (0.13) | 0.83 (0.04) | 0.87413 |
|  |  | Roundness^1^ | 0.77 (0.10) | 0.71 (0.08) | **3.00E-08** |
|  |  | Solidity^1^ | 0.93 (0.04) | 0.95 (0.01) | **0.00048** |
|  | Structure | Fractal Dimension Index^2^ | 1.83 (0.05) | 1.78 (0.01) | 0.22713 |
| *C. tropicalis* V546 | Size | Length, µm | 2.16 (0.44) | 2.29 (0.47) | **0.0065** |
|  |  | Cell area, µm^2^ | 3.66 (1.38) | 4.00 (1.32) | **0.02348** |
|  | Form | Aspect ratio (AR), width/length | 1.33 (0.15) | 1.41 (0.17) | **2.30E-05** |
|  |  | Elongation, 1- AR | -0.33 (0.15) | -0.41 (0.17) | **2.30E-05** |
|  | Shape | Perimeter, µm | 7.20 (1.40) | 7.62 (1.28) | **0.00548** |
|  |  | Circularity^1^ | 0.86 (0.05) | 0.84 (0.05) | **0.00026** |
|  |  | Roundness^1^ | 0.76 (0.08) | 0.72 (0.08) | **2.40E-05** |
|  |  | Solidity^1^ | 0.93 (0.02) | 0.93 (0.02) | 0.48658 |
|  | Structure | Fractal Dimension Index^2^ | 1.85 (0.03) | 1.81 (0.01) | 0.19784 |
| *S. aureus*  ATCC 25923 | Size | Length, µm | 0.70 (0.08) | 0.79 (0.11) | **4.10E-14** |
|  |  | Cell area, µm^2^ | 0.38 (0.08) | 0.45 (0.08) | **1.00E-11** |
|  | Form | Aspect ratio (AR), width/length | 1.18 (0.12) | 1.20 (0.14) | 0.1773 |
|  |  | Elongation, 1- AR | -0.18 (0.12) | -0.20 (0.14) | 0.1773 |
|  | Shape | Perimeter, µm | 2.35 (0.28) | 2.71 (0.39) | **4.50E-16** |
|  |  | Circularity^1^ | 0.86 (0.06) | 0.79 (0.12) | **3.30E-06** |
|  |  | Roundness^1^ | 0.85 (0.07) | 0.84 (0.09) | 0.17988 |
|  |  | Solidity^1^ | 0.93 (0.02) | 0.91 (0.05) | **0.00429** |
|  | Structure | Fractal Dimension Index^2^ | 1.80 (0.04) | 1.65 (0.01) | **0.01878** |
| *S. aureus*  MRSA 333 | Size | Length, µm | 0.73 (0.08) | 0.71 (0.16) | 0.03122 |
|  |  | Cell area, µm^2^ | 0.40 (0.06) | 0.37 (0.14) | **7.50E-05** |
|  | Form | Aspect ratio (AR), width/length | 1.20 (0.13) | 1.27 (0.22) | **0.00556** |
|  |  | Elongation, 1- AR | -0.20 (0.12) | -0.27 (0.21) | **0.00556** |
|  | Shape | Perimeter, µm | 2.45 (0.23) | 2.52 (0.69) | 0.13391 |
|  |  | Circularity^1^ | 0.84 (0.07) | 0.74 (0.16) | **7.00E-14** |
|  |  | Roundness^1^ | 0.84 (0.08) | 0.80 (0.11) | **0.00543** |
|  |  | Solidity^1^ | 0.93 (0.02) | 0.91 (0.08) | 0.10223 |
|  | Structure | Fractal Dimension Index^2^ | 1.80 (0.01) | 1.95 (0.01) | **0.00099** |
| *K. pneumoniae*  ATCC 33495 | Size | Length, µm | 0.96 (0.28) | 0.94 (0.27) | 0.9846 |
|  |  | Cell area, µm^2^ | 0.58 (0.21) | 0.62 (0.29) | 0.34904 |
|  | Form | Aspect ratio (AR), width/length | 1.69 (0.43) | 1.52 (0.36) | **0.00083** |
|  |  | Elongation, 1- AR | -0.68 (0.43) | -0.52 (0.36) | **0.00083** |
|  | Shape | Perimeter, µm | 3.28 (0.73) | 3.28 (0.85) | 0.9586 |
|  |  | Circularity^1^ | 0.67 (0.12) | 0.70 (0.11) | **0.03048** |
|  |  | Roundness^1^ | 0.63 (0.15) | 0.69 (0.15) | **0.00085** |
|  |  | Solidity^1^ | 0.89 (0.05) | 0.89 (0.05) | 0.1181 |
|  | Structure | Fractal Dimension Index^2^ | 1.75 (0.07) | 1.86 (0.04) | 0.08739 |
| *K. pneumoniae*  KPC 609803 | Size | Length, µm | 1.63 (0.57) | 1.45 (0.43) | 0.08345 |
|  |  | Cell area, µm^2^ | 1.15 (0.48) | 1.04 (0.37) | 0.16041 |
|  | Form | Aspect ratio (AR), width/length | 2.69 (1.30) | 2.28 (0.80) | **0.03638** |
|  |  | Elongation, 1- AR | -1.69 (1.30) | -1.28 (0.79) | **0.03638** |
|  | Shape | Perimeter, µm | 5.65 (1.50) | 5.64 (1.52) | 0.89358 |
|  |  | Circularity^1^ | 0.47 (0.16) | 0.44 (0.15) | 0.08977 |
|  |  | Roundness^1^ | 0.47 (0.23) | 0.49 (0.18) | **0.03602** |
|  |  | Solidity^1^ | 0.82 (0.11) | 0.79 (0.10) | **0.00077** |
|  | Structure | Fractal Dimension Index^2^ | 1.66 (0.03) | 1.86 (0.04) | **0.00382** |

**Legend:** ^1^ Shape descriptor/parameter calculated through a classification between 0 and 1. ^2^ Values of fractal dimension index between control and honey-treated samples were compared through Student’s *t*-test. ^3^ Pairwise comparisons were realized through the Wilcoxon test. Evaluation of the *in vitro* antimicrobial effect of honey samples on cell morphological parameters in 24-hour biofilms through SEM analysis. Parametric *t*-tests and non-parametric Wilcoxon tests were used to identify significant differences (*P*-values <0.05). The results of the analysis presented in this table were performed in Fiji-ImageJ (version 1.57).
